# Tryptophan metabolism at the crossroads of the neuro-immuno-microbial axis: implications for precision medicine in chronic diseases

**DOI:** 10.3389/fcimb.2025.1707850

**Published:** 2026-01-12

**Authors:** Huang-Hui Xia, Jian-Zhong Huang

**Affiliations:** 1 College of Life Sciences, Fujian Normal University, Fuzhou, China; 2 Engineering Research Center of Industrial Microbiology, Ministry of Education, National and Local United Engineering Research Center of Industrial Microbiology and Fermentation Technology, Fujian Normal University, Fuzhou, China

**Keywords:** gut-microbiota-brain axis, kynurenine pathway, neuro-immuno-microbial axis, precision medicine, tryptophan metabolism

## Abstract

Tryptophan metabolism connects the nervous, immune, and microbial systems and influences the onset and progression of chronic diseases such as cancer, neurodegeneration, and autoimmunity. This review summarizes the three major metabolic routes: the kynurenine pathway, the serotonin pathway, and microbial indole production. It outlines how their metabolites shape neural activity, immune regulation, and host–microbiota interactions. We further discuss the relevance of these metabolites as biomarkers and their potential therapeutic implications. By integrating recent insights into tryptophan-associated signaling networks, this review provides a concise framework for understanding their roles in chronic disease and guiding future precision medicine strategies.

## 1 Introduction

Tryptophan is an essential amino acid required for protein synthesis and for maintaining metabolic and immune homeostasis (Zheng et al., 2025). Unlike plants and microorganisms, mammals cannot synthesize tryptophan *de novo* and must obtain it from the diet. In humans, dietary tryptophan is mainly metabolized through three pathways: the kynurenine pathway, the serotonin pathway, and microbial indole production (Roager and Licht, 2018; Madella et al., 2022; El-Azaz et al., 2023). Its metabolic fate is shaped not only by dietary intake but also by competition with other amino acids, gut microbial activity, and interactions with other nutrients.

**Figure 1:**
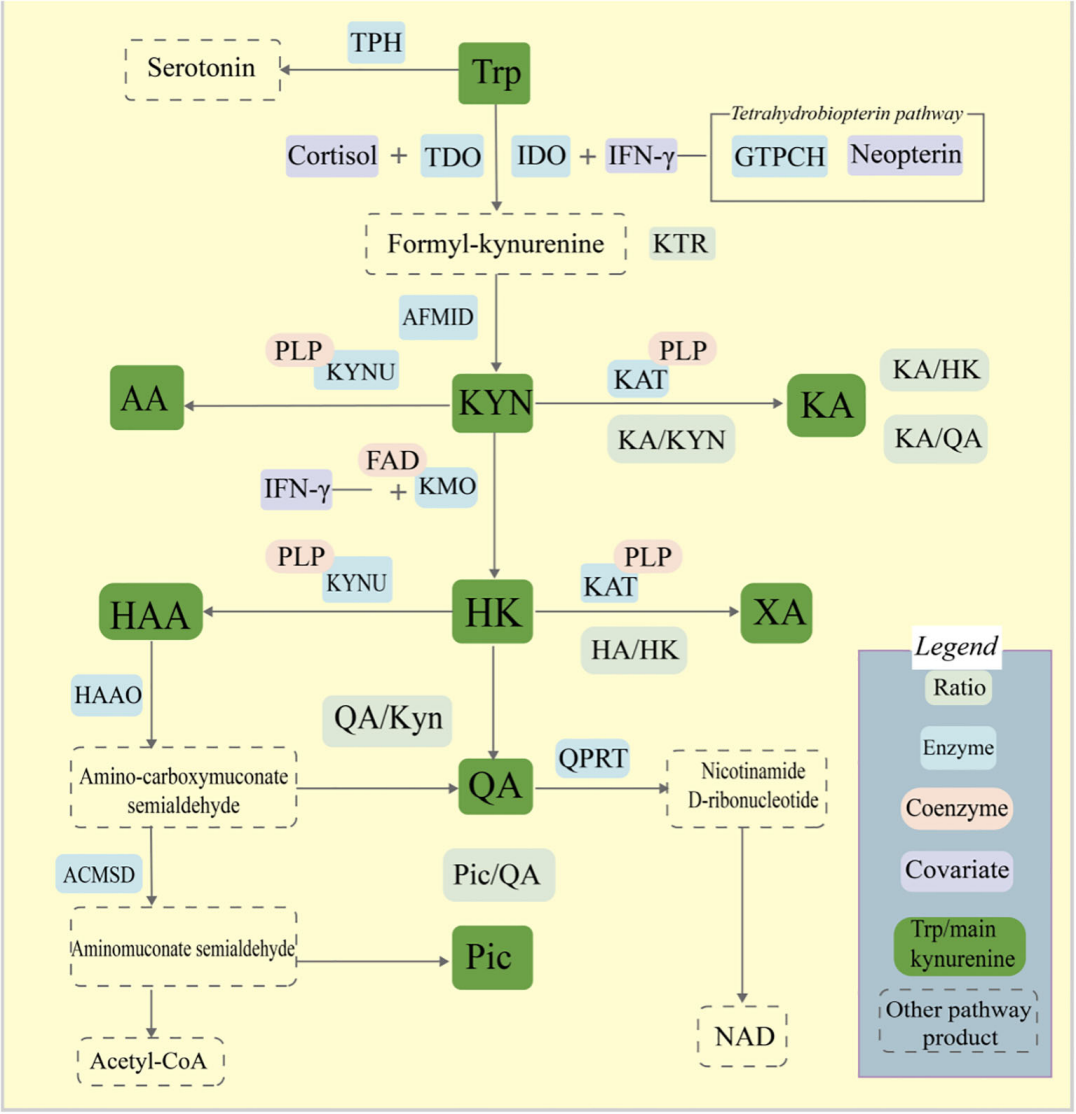
The kynurenine pathway of tryptophan metabolism. Key enzymes, metabolites, and cofactors involved in the pathway are shown. 3-HAA, 3-hydroxyanthranilic acid; 3-HK, 3-hydroxykynurenine; AA, anthranilic acid; ACMSD, aminocarboxymuconate semialdehyde decarboxylase; AHR, aryl hydrocarbon receptor; IDO, indoleamine 2,3-dioxygenase; KA, kynurenic acid; KAT, kynurenine aminotransferase; KMO, kynurenine monooxygenase; KYN, kynurenine; KYNU, kynureninase; NAD+, nicotinamide adenine dinucleotide; Pic, picolinic acid; QA, quinolinic acid; QPRT, quinolinate phosphoribosyltransferase; TDO, tryptophan 2,3-dioxygenase; Tryptophan, tryptophan; XA, xanthurenic acid.

**Figure 2:**
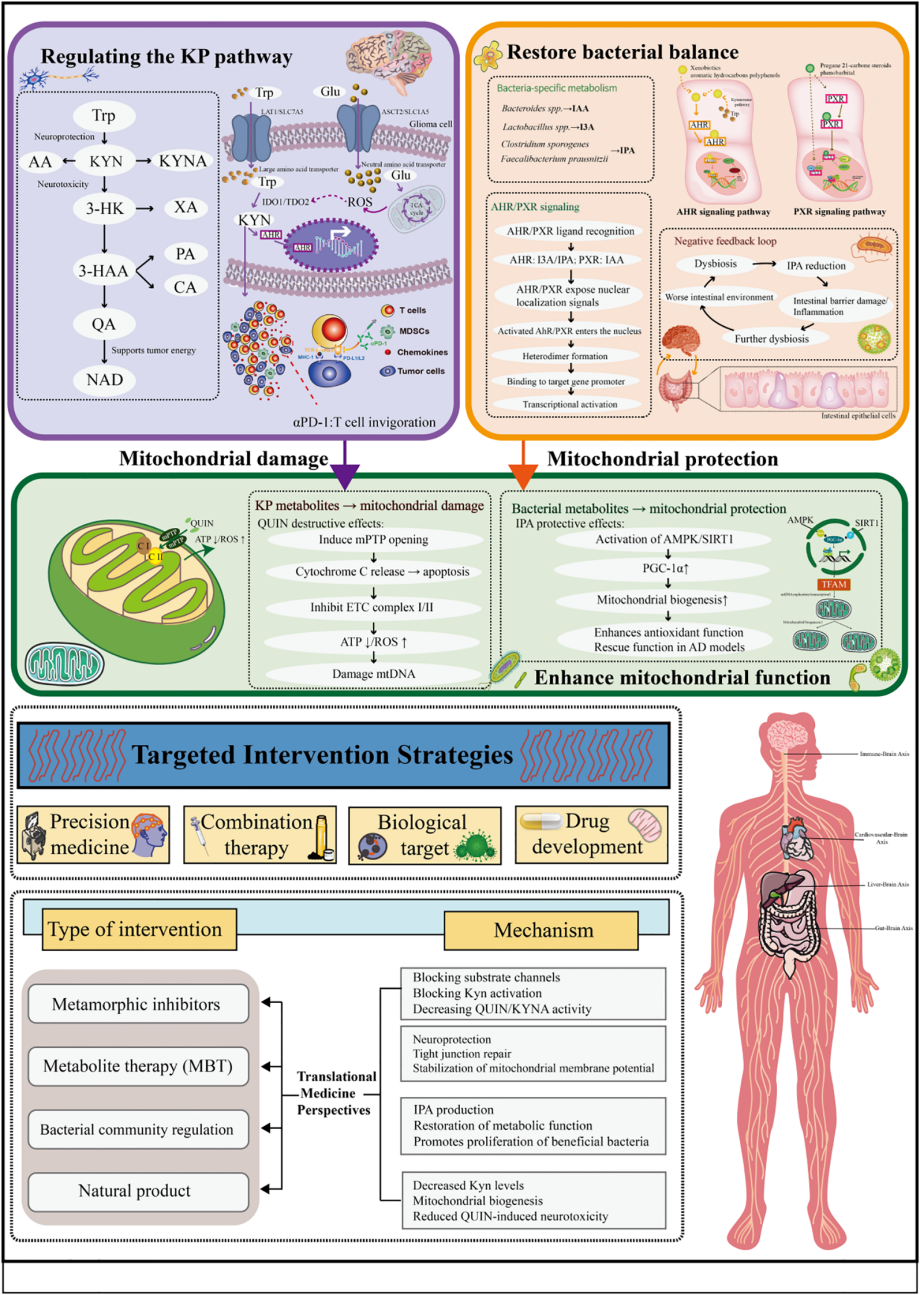
Overview of kynurenine pathway, intestinal flora, and mitochondrial function.

The kynurenine pathway accounts for most tryptophan degradation, while the serotonin pathway and microbial indole production contribute additional metabolic outputs (**Figure 1**) (Savitz, 2020; Tanaka et al., 2021; Tanaka et al., 2022). Interactions among these pathways link metabolic regulation with immune responses and neural function. Commensal microbes further diversify tryptophan metabolism by generating indole derivatives such as indole-3-acetic acid (IAA), indole-3-carbaldehyde (I3A), and tryptamine. Several of these metabolites have emerging value as biomarkers for disease stratification and therapy monitoring. Tryptophan metabolism connects the nervous, immune, and microbial systems, influencing the development and progression of chronic diseases such as cancer, neurodegenerative disorders, and autoimmune conditions (**Figure 2**) (Cellini et al., 2020; Mondanelli et al., 2020; Bellucci et al., 2021; Mandarano et al., 2021).

A better understanding of its regulation is essential for developing targeted therapies. Emerging strategies, such as enzyme inhibitors, receptor modulators, and microbiota-targeted therapies, offer potential solutions for precision medicine in chronic diseases. Future research should focus on refining these therapeutic approaches and exploring novel strategies to restore metabolic balance. This review synthesizes how these pathways form a neuro–immune–microbial axis and how their dysregulation contributes to chronic disease.

## 2 Molecular mechanisms and signaling hubs

Dysregulation of tryptophan metabolism has been implicated in the development of chronic diseases, making its enzymes, metabolites, and receptors potential therapeutic targets. Key metabolites of the kynurenine pathway (KP), such as kynurenine (KYN), act as agonists for the aryl hydrocarbon receptor (AHR).

KP metabolites have dual effects on glutamatergic neurotransmission. They specifically target the N-methyl-D-aspartate receptor(NMDAR). These metabolites can exert either neuroprotective effects or neurotoxic effects. Dysregulation of this pathway is intimately linked to multiple neuropsychiatric and neurological disorders, including schizophrenia, major depressive disorder, cognitive impairment, neuropathic pain, and neurodegenerative diseases (Jovanovic et al., 2020; Muneer, 2020). For example, inhibiting the key enzyme kynurenine 3-monooxygenase(KMO) promotes the metabolic flux toward the neuroprotective kynurenic acid(KYNA), which effectively alleviates neuropathic pain and its accompanying depressive-like behavior in preclinical models. Likewise, a pathological shift within the KP toward the neurotoxic metabolite 3-hydroxykynurenine(3-HK) is implicated in glaucomatous neurodegeneration, highlighting the potential of KMO inhibition as a novel therapeutic strategy (Fiedorowicz et al., 2021). Notably, the active form of vitamin B_6_, pyridoxal 5’-phosphate(PLP)—an essential cofactor for multiple tryptophan-metabolizing enzymes—represents a critical regulator of metabolic crosstalk at the host-microbiome interface (Cellini et al., 2020).

Microbial metabolism of tryptophan constitutes a major axis through which the gut microbiota influences host health. Tryptophan-derived microbial metabolites modulate intestinal barrier integrity, immune homeostasis, and neural activity, primarily via activation of pathways such as the AHR (Roth et al., 2021). Dietary supplementation with tryptophan or its precursors has been demonstrated to ameliorate symptoms of inflammatory bowel disease(IBD), enhance sleep quality, improve mood states, and may potentially prevent hypertension in offspring induced by maternal chronic kidney disease(CKD), likely through modulation of the microbiota-metabolite-AHR axis (Hsu et al., 2020). This paradigm offers novel therapeutic avenues for targeted modulation of tryptophan metabolism to promote health, utilizing probiotics, prebiotics, and postbiotics.

### 2.1 Fine regulation of the kynurenine pathway

**Figure 3:**
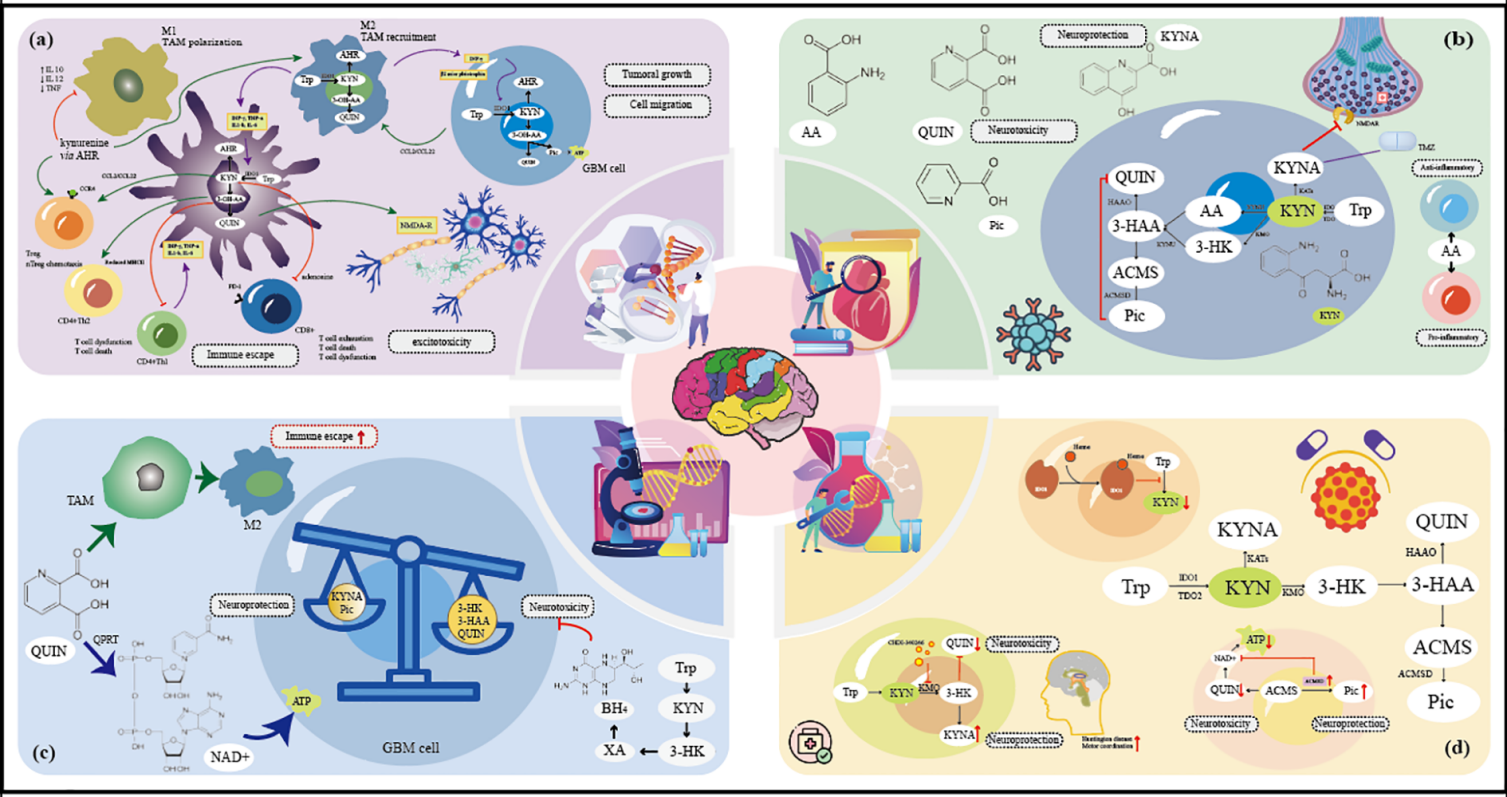
Panorama of metabolic regulation and therapeutic targets of the KP in gliomas. **(a)** Mechanisms of aberrant activation of the KP in the glioma microenvironment; **(b)** Mechanisms of metabolic flow and neuroprotective-neurotoxicity balance regulated by enzyme activity at the midstream branching point of the KP; **(c)** Positive-negative balance of KP metabolites in gliomas tilt mechanism; **(d)** therapeutic strategies to target KP fine-tuning nodes.

The KP is the primary route for tryptophan degradation and plays a critical role in glioma progression. In gliomas, abnormal activation of the KP supports tumor growth by providing energy and biosynthetic resources through metabolic reprogramming. Moreover, the KP generates immunosuppressive metabolites that contribute to tumor progression and immune evasion (**Figure 3a**) (Ha et al., 2014; Thakur et al., 2020).

#### 2.1.1 Impact of dysregulation in gliomas

One key regulatory step in the kynurenine pathway is the uptake of tryptophan and kynurenine (KYN) via transporters such as L-type amino acid transporter(LAT1) and ASCT2, which are often overexpressed in glioma. Targeting these transporters to deprive tumors of essential substrates offers a potential strategy to inhibit tumor growth (Bröer et al., 2016; Cormerais et al., 2018). The subsequent initial oxygenation step, catalyzed by the key rate-limiting enzymes IDO1 and TDO2, converts Tryptophan to L-KYN. IDO1 expression exhibits significant heterogeneity across glioma subtypes (Sordillo et al., 2017), positively correlates with increasing tumor grade. It is modulated by isocitrate dehydrogenase (IDH) mutation status, which showing marked upregulation in IDH-wildtype tumors (Rocha et al., 2025). TDO2 demonstrates constitutive overexpression in high-grade gliomas, particularly glioblastoma(GBM), serving as an independent biomarker for tumor aggressiveness and poor prognosis (Riess et al., 2020; Wang et al., 2025). Critically, KYN produced by TDO2 directly activates the AHR as a ligand, driving tumor cell proliferation, invasion, and stem-like properties (Adams et al., 2012). Consequently, IDO1 inhibitors enhance anti-tumor immunity and show strong synergy when combined with immune checkpoint blockade (Guangzhao et al., 2025). Furthermore, post-translational modifications (PTMs)—such as phosphorylation and acetylation—have been demonstrated to dynamically regulate IDO1 stability and function, while enhancing TDO2 enzymatic activity, thereby providing an additional layer of fine control over KP flux initiation.

As outlined in Part 1, L-KYN sits at a critical branch point, destined for conversion into either neuroprotective or neurotoxic metabolites. Here, we focus on how glioma-specific enzyme expression alters this balance (**Figure 3b**). Kynurenine aminotransferases (KATs) catalyze the irreversible transamination of L-KYN to generate kynurenic acid(KYNA). Beyond its role as a potent NMDAR antagonist, KYNA exerts direct anti-tumor effects by suppressing glioma cell proliferation and migration, and exhibits synergy with chemotherapeutics like temozolomide (TMZ) (Badawy, 2017). Conversely, KMO catalyzes the conversion to 3-HK, which is subsequently metabolized into neurotoxic metabolites—3-hydroxyanthranilic acid (3-HAA) and quinolinic acid (QA) (Venkatesan et al., 2020). KMO is markedly overexpressed and enzymatically hyperactive in astrocytomas. Its metabolites promote tumor progression and exacerbate neuronal injury through three convergent mechanisms: glutamate receptor hyperactivation, induction of oxidative stress, and DNA damage. Inhibiting KMO effectively redirects metabolic flux toward KYNA production, alleviating neuropathological symptoms and inhibiting tumors in preclinical models (Dudzinska et al., 2019).

In gliomas, the metabolite balance shifts toward neurotoxicity and tumor progression, with a decrease in protective metabolites and an increase in neurotoxic or oncogenic species (**Figure 3c**) (Moffett et al., 1993)le prognostic indicator (Krupa et al., 2025). AA accumulates in gliomas and contributes to disease progression through its context-dependent pro- and anti-inflammatory properties. Meanwhile, the enzyme ACMSD Kynureninase(KYNU) catalyzes the production of anthranilic acid(AA), whose high expression is significantly associated with a shorter overall survival in glioma patients and is a reliabacts as a critical “molecular switch” by diverting its substrate(ACMS) toward picolinic acid(PIC) and away from the neurotoxic metabolite quinolinic acid(QUIN) (Brundin et al., 2016; Fargher et al., 2025).

QUIN promotes tumors through two mechanisms: it converts to NAD^+^ via quinolinic acid phosphoribosyltransferase (QPRT), providing glioma cells with energy and substrates for DNA repair, thus supporting survival and proliferation; additionally, QUIN induces tumor-associated macrophages (TAMs) to polarize toward the immunosuppressive M2 type, aiding immune evasion (Wang et al., 2010; Basak et al., 2023; Wang et al., 2025). In addition, xanthurenic acid(XA), a downstream metabolite of 3-HK, can inhibit tetrahydrobiopterin(BH_4_) synthesis, interfere with neurotransmitter balance, and exhibit a unique inhibitory effect on tumor cell motility in GBM (Staats Pires et al., 2020). This metabolic imbalance results from upregulated activity of tumor-promoting enzymes (IDO1, TDO2, KMO, KYNU) alongside downregulated protective enzymes(KATs, ACMSD). Downregulation of ACMSD expression, commonly observed in gliomas, leads to a significant shift in metabolic flux toward QUIN and nicotinamide adenine dinucleotide (NAD^+^) synthesis pathways (Moffett et al., 2020; Dhuguru et al., 2023; Huang et al., 2025).

#### 2.1.2 Therapeutic implications for targeting KP

Therapies targeting KP nodes are being developed. IDO1 inhibitors reduce KYN and T cell exhaustion, restoring anti-tumor immune responses (**Figure 3d**) (Kang et al., 2025; Li et al., 2025). In addition, dual IDO/TDO inhibitors can simultaneously target IDO1 and TDO2, overcoming compensatory upregulation after single enzyme inhibition, such as HIF-1α-mediated TDO2 escape phenomena (Wu et al., 2023). Targeting KP branch enzymes can restore the neuroprotective-neurotoxic balance. Inhibition of KMO with CHDI-340246 shifts metabolism from QUIN to KYNA, thus ameliorating motor deficits in HD models (Beaumont et al., 2016). The strategy of restoring or expressing ACMSD function can effectively reduce the cyclization of ACMS to QUIN, reduce the production of neurotoxic metabolites, and inhibit the energy supply of the tumor (Tufail et al., 2024). Further strategies aim to antagonize toxic metabolites directly. Inhibiting quinolinic acid phosphoribosyltransferase (QPRT) with compounds like UPF648 blocks QUIN’ s conversion to NAD^+^. Moreover, co-inhibiting QPRT and NAMPT (e.g., with FK866) synergistically blocks NAD^+^ synthesis, inducing lethal energy depletion in tumor cells and enhancing therapeutic efficacy (Kudo et al., 2024). Multi-target drug design addresses KP complexity. The dual inhibitor RY103, which targets both IDO1/TDO2 and the KYN-AHR-AQP4 axis, has been shown to suppress tumor invasion and migration in GBM models (Liang et al., 2021). To address the compensatory mechanisms and multifunctionality of the KP network—which often render single-agent therapies ineffective—combination strategies targeting multiple nodes are being pursued to enhance efficacy and overcome resistance (Garg et al., 2024). Thus, the multi-target therapeutic strategies discussed herein have shown promise in advancing neuro-oncological treatment for gliomas, and early-stage research suggests their potential application in other conditions involving KP dysregulation.

### 2.2 Interaction mechanism between the microbiota and tryptophan axis

#### 2.2.1 Microbiota’s role in tryptophan metabolism

The gut microbiota plays a pivotal role in regulating tryptophan metabolism, influencing both host and microbial interactions. Beyond the host-driven kynurenine and serotonin pathways, microbial metabolism of tryptophan generates indole derivatives that are essential for maintaining intestinal barrier integrity, immune function, and neurological health. Approximately 1-5% of dietary Tryptophan is metabolized by specific bacterial communities in the colon, producing a variety of structurally diverse indole compounds. The production of these metabolites exhibits significant species dependence (Jamshed et al., 2022). For example, *Bacteroides* spp. primarily produce IAA, while *Lactobacillus acidophilus*, *Lactobacillus murinus*, and *Lactobacillus reuteri* produce I3A (Taleb, 2019; Scott et al., 2020). Spore-forming bacteria like *Clostridium* sp*orogenes* synthesize indolepropionic acid(IPA) (Peng et al., 2024). Indole derivatives from microorganisms exert local effects in the intestine, but metabolites like IPA and I3A can also cross the blood-brain barrier(BBB) and impact the central nervous system (Hu et al., 2025).

#### 2.2.2 Gut microbiota and neuroactive effects

The neuroactive effects of tryptophan metabolites depend largely on whether they can cross the BBB. Because the BBB tightly controls molecular exchange, only metabolites that can traverse this interface exert direct effects in the central nervous system. The transport mechanisms vary significantly: some metabolites rely on specific active transporters, while others passively diffuse, and this differential access critically shapes their respective roles in neurological health and disease. The neuroactive effects of these KP metabolites are contingent upon their distinct interactions with the BBB. The key metabolite, L-KYN, crosses the blood-brain barrier (BBB) through LAT1, the same transporter it shares with its precursor, Tryptophan (Hsu et al., 2020). This access allows for the local production of downstream metabolites within the brain itself. For instance, once across the BBB, L-KYN can be converted by astrocytic kynurenine aminotransferases (KATs) to form KYNA, which, being a polar organic acid, has limited back-diffusion and thus accumulates locally. In contrast, the neurotoxic metabolite QUIN is transported across the BBB by an active, saturable system, leading to its accumulation under inflammatory conditions when peripheral production is high. Conversely, the neuroprotective KYNA has limited permeability, underscoring the importance of its *in situ* synthesis within the CNS for its neuromodulatory functions.

#### 2.2.3 Impact on AHR activation and gut health

Microbial indoles engage the AHR pathway to regulate epithelial integrity and immune tolerance, as summarized in the general mechanism. Here we focus on their disease-specific consequences. The absence of specific probiotics, especially those that produce IPA(such as *Faecalibacterium prausnitzii*) and I3A-producing *lactobacilli*, leads to depletion of protective metabolites, resulting in decreased levels of IPA and I3A, insufficient AHR activation, weakened intestinal barrier function, and exacerbated immune imbalance (Huang et al., 2023; Xu et al., 2024). In addition, pro-inflammatory factors released by intestinal inflammation inhibit the growth of probiotics while upregulating the expression of host IDO1, accelerating the degradation of Tryptophan along the KP and further reducing the substrate of Tryptophan metabolized into indole derivatives in the microbiota (Kaur et al., 2025). As inflammation worsens, oxidative stress and bile acid changes also inhibit the colonization of indole-producing bacteria. The lack of IPA not only damages the local immune defense of the intestine but also inhibits its ability to cross the blood-brain barrier, leading to insufficient activation of AHR in the central nervous system and thereby affecting the anti-inflammatory response of astrocytes. For example, in neuroinflammatory models such as MS, IPA deficiency promotes disease progression (Zelic et al., 2021). Clinical studies have shown that IPA levels in IBD patients are positively correlated with cognitive function scores (Sârb et al., 2025), further confirming the pivotal role of the microbiota-Tryptophan metabolic axis in gut-brain communication.

However, in chronic disease states, the ability of gut microbiota to metabolize tryptophan and produce AHR ligands is significantly impaired. Studies have shown that fecal AHR agonist activity and the concentrations of key indole ligands such as IAA are significantly reduced in patients with metabolic syndrome (MetS) and patients in remission of inflammatory bowel disease (IBD) (Lamas et al., 2016). This deficiency is not only related to dysbiosis of gut microbiota composition, but is also directly regulated by the host’s genetic background. For example, the deletion of the IBD susceptibility gene CARD9 alters the gut microbiota, leading to a reduction in AHR ligand-producing bacteria (such as Lactobacillus reuteri), thereby weakening AHR signaling and increasing susceptibility to colitis (Niu et al., 2025). This indicates that disruption of the “host gene-microbiota function-AHR ligand production” axis is a common pathological basis for many chronic diseases.

#### 2.2.4 Dysbiosis and disease pathogenesis

Based on the above mechanism, intervention strategies targeting the microbiota-tryptophan axis show great therapeutic potential. Supplementing with probiotics that produce indole metabolites or directly administering indole derivatives can effectively restore AHR/PXR activation and improve intestinal barrier damage and inflammatory responses in IBD models. In addition, increasing dietary Tryptophan intake or using AHR agonists can reverse neurodeficiencies associated with dysbiosis, especially in AD models, where IPA exerts neuroprotective effects through the CPR30/AMPK/SIRT1 pathway and has antioxidant effects (Rakshe et al., 2024). Short-chain fatty acids, such as butyric acid, also indirectly promote the diversion of Tryptophan to indole metabolism in the microbiota by inhibiting IDO1 expression and STAT1 phosphorylation, further enhancing the AHR signaling pathway (Pant et al., 2023).

In summary, the microbiota-tryptophan axis regulates the AHR/PXR signaling network through microbial-derived indole metabolites, serving as a core regulatory mechanism for intestinal barrier defense and immune balance. The disruption of this axis, such as dysbiosis, indole deficiency, AHR inhibition, barrier damage, immune imbalance, exacerbated inflammation, and worsening microbiota, is a common pathological basis for many chronic diseases. Restoring homeostasis within the tryptophan metabolic network may offer potential targets for intervention in cross-system diseases, though further research is needed to confirm their applicability across various conditions.

### 2.3 The synergistic network of microbial metabolites: integrating short-chain fatty acids and tryptophan derivatives

#### 2.3.1 Short-chain fatty acids and their immunomodulatory effects

The impact of the gut microbiota on host physiology is mediated by a complex network of metabolites, among which short-chain fatty acids (SCFAs) and tryptophan-derived indoles represent two pivotal communicative channels. While often studied in parallel, their functions are deeply intertwined, creating a synergistic system that maintains host homeostasis.

SCFAs are produced by microbial fermentation of dietary fiber and exert profound immunomodulatory effects. A key mechanism is their function as histone deacetylase(HDAC) inhibitors, which epigenetically regulates gene expression in immune cells (Qu et al., 2023). For instance, SCFAs, particularly butyrate and propionate, promote the differentiation of IL-10-producing regulatory B cells (Bregs) and have been shown to alleviate disease severity in models of rheumatoid arthritis by this mechanism. Furthermore, SCFAs are crucial for B cell metabolism, fueling their activation and antibody production through enhanced glycolysis, oxidative phosphorylation, and fatty acid synthesis.

#### 2.3.2 Synergistic effects between SCFAs and tryptophan derivatives

The interplay between SCFAs and the tryptophan-AHR axis is multifaceted. SCFAs can indirectly promote the tryptophan-indole pathway by inhibiting the expression of the host’s IDO1 enzyme, thereby increasing the bioavailability of tryptophan for gut microbes (Yi et al., 2025). Conversely, AHR activation by indole derivatives can enhance the integrity of the gut barrier, an environment that SCFAs also help to maintain. This creates a positive feedback loop: a healthy barrier supports a SCFA-producing microbiota, which in turn shunts more tryptophan toward the production of AHR ligands that further fortify the barrier.

This metabolic cross-talk is evident in disease pathogenesis. Dysbiosis often leads to a concurrent deficiency in both SCFAs and AHR ligands. For example, in metabolic syndrome, the observed decline in microbial AHR agonist production occurs alongside broader microbial disturbances (Natividad et al., 2018). Therapeutic strategies that simultaneously target both metabolite families—such as a diet rich in both fermentable fiber(for SCFAs) and tryptophan(for indoles)—or next-generation probiotics designed to produce both SCFAs and indoles, may yield superior efficacy in restoring immune and metabolic homeostasis in chronic diseases ranging from IBD to metabolic syndrome.

### 2.4 Mitochondrial-metabolite crosstalk

#### 2.4.1 Impact of tryptophan metabolites on mitochondrial function

Tryptophan metabolites from the KP and microbiota-derived indoles influence mitochondria both as metabolic regulators and as direct modulators of mitochondrial structure and signaling. By affecting survival, apoptosis, and autophagy, they help determine cellular energy balance. This metabolit mitochondria interaction is central to neurodegeneration, tumor metabolic reprogramming, and inflammation. Metabolites of the canine urea cycle, especially QUIN, are potent neurotoxic metabolites that directly interfere with mitochondrial function, causing dysfunction and cell damage (Platten et al., 2021).

#### 2.4.2 Role of QUIN and KYNA in neurodegenerative diseases

QUIN promotes the generation of significant levels of reactive oxygen species (ROS) and interferes with intracellular calcium balance, resulting in an increased permeability of the mitochondrial membrane. QUIN acts as a potent redox cycler and a direct inhibitor of the mitochondrial electron transport chain (ETC), particularly at complexes I and II (Zhao et al., 2019). This inhibition causes a substantial leak of electrons, which reduce molecular oxygen to generate superoxide anions (O_2_^-^), the primary mitochondrial ROS. The resulting oxidative damage further impairs ETC function, creating a vicious cycle of ROS production. Concurrently, QUIN depletes glutathione (GSH) and inhibits antioxidant enzymes like superoxide dismutase (SOD), crippling the cellular defense system and amplifying the oxidative insult (Bansal et al., 2019; Krishnamurthy et al., 2024). The disruption of calcium homeostasis is a hallmark of QUIN toxicity. By acting as an agonist of N-methyl-D-aspartate receptors (NMDARs), QUIN triggers excessive Ca^2+^ influx into neurons. The resultant cytosolic Ca^2+^ overload is rapidly buffered by mitochondria, leading to mitochondrial Ca^2+^ overload (Madreiter-Sokolowski et al., 2020). This overload is a critical trigger for the prolonged opening of the mitochondrial permeability transition pore (mPTP). Sustained mPTP opening collapses the mitochondrial membrane potential (ΔΨm), uncouples oxidative phosphorylation, and causes osmotic swelling, outer membrane rupture, and the release of pro-apoptotic factors such as cytochrome c, thereby initiating caspase-dependent apoptosis. This mechanism may play a significant role in neurodegenerative conditions such as Huntington’s disease (HD) and Alzheimer’s disease (AD), as well as in ischemia-reperfusion injury, based on current evidence, though further studies are needed to fully establish its involvement (Wadan et al., 2025).

In contrast, the neuroprotective metabolite KYNA exhibits antioxidant properties and can partially antagonize QUIN-induced mitochondrial oxidative stress and dysfunction (Saliba et al., 2025). By inhibiting KMO, which promotes metabolic flux toward KYNA production, it has been shown in preclinical models to protect mitochondria and reduce neurological damage (Beggiato et al., 2014).

#### 2.4.3 Microbial metabolites and mitochondrial health

Microbial-derived indole metabolites, especially IPA, exhibit significant mitochondrial protection and metabolic regulation effects. IPA enhances mitochondrial biogenesis by activating the peroxisome proliferator-activated receptor gamma coactivator 1α (PGC-1α) pathway (Li et al., 2025). IPA, as a potent inducer of PGC-1α, can significantly upregulate the expression and activity of PGC-1α by activating AMP-dependent protein kinase(AMPK) and silencing the deacetylase regulatory protein(SIRT1) (Majeed et al., 2021). Activated PGC-1α works synergistically with nuclear respiratory factors (NRF-1/2) and mitochondrial transcription factor A(TFAM) to drive mitochondrial DNA replication, ETC complex protein expression, and new mitochondrial generation, thereby enhancing the cell’s oxidative phosphorylation capacity and energy production (Liang et al., 2020). In addition, IPA promotes mitophagy by activating the SIRT1-PGC-1α axis, clearing damaged mitochondria and enhancing the expression of antioxidant enzymes, effectively alleviating oxidative damage to mitochondria and maintaining their healthy state (Liang et al., 2020). IPA also regulates the activity of key metabolic enzymes by optimizing substrate utilization and respiratory efficiency, promoting fatty acid *β*-oxidation and glucose oxidation, ensuring efficient substrate flow into the tricarboxylic acid cycle(TCA cycle), and supporting the efficient operation of the ETC (Liu et al., 2025).

In summary, understanding how tryptophan metabolites interact with mitochondria offers a new approach to treating related diseases (**Table 1**). By inhibiting the production of toxic metabolites, using KMO inhibitors, enhancing ACMSD activity, reducing QUIN levels, and promoting the accumulation of neuroprotective KYNA, mitochondria can be protected from excitotoxicity and oxidative damage. Additionally, supplementing with beneficial microbiota metabolites like IPA or using IPA-producing probiotics can activate the PGC-1α pathway, improving mitochondrial function. This is particularly important for treating neurodegenerative diseases and metabolic syndrome.

**Table 1:** Multidimensional regulatory mechanisms and translational applications of the tryptophan metabolic network in diseases.

Metabolites	Source and regulation	Molecular targets	Pathway	Physiological functions	Disease associations	Mechanism	References
IPA	Source: •C. sporogenes • F. prausnitzii Regulates: • Dietary fiber ↑→ production ↑ • Intestinal inflammation ↓→ production ↑	• AhR/PXR • PGC-1/AMPK/SIRT1	1. AhR→ZO-1/occludin↑→Barrier repair 2. PXR→NF-κB↓→inflammation suppression 3. AMPK/SIRT1→PGC-1α↑→mitochondrial biogenesis↑ 4. AhR→PPARγ↑→Treg differentiation↑(IL-10↑)	•Maintaining the integrity of intestinal epithelial tight junctions •inhibitionTh17differentiation •Crossing the blood-brain barrier •Activate astrocytesAhR→neuroinflammation↓ •Enhanced mitophagy	IBD Type 2 diabetes MS AD	•Intestinal barrier damage→Endotoxin translocation↑ •neuroinflammation↑→Microglial activation↑	(Pant et al., 2023; Rakshe et al., 2024; Hu et al., 2025; Sârb et al., 2025)
Indole-3-aldehyde (I3A)	Sources: • Lactobacillus reuteri • Lactobacillus acidophilus Regulation: • Tryptophanase-positive bacteria colonization ↑ → yield ↑ • Intestinal hypoxia → yield ↓	AhR	1. AhR → ILC3 activation → IL-22↑ → RegIIIγ↑ 2. AhR → STAT3↓ → Th17 differentiation↓ 3. AhR → mucin (MUC2)↑	•Strengthening the intestinal chemical barrier (Antimicrobial peptide secretion) •Maintain goblet cell function •PromoteIL-22Dependent epithelial repair	IBD Autoimmune hepatitis	•Thinning of the mucus layer→Pathogen invasion↑ • Th17/Treg imbalance → tissue damage↑	(Peng et al., 2024)
Quinolinic acid (QUIN)	Source: •HostIDO1/TDO (Inflammation induction) Regulation: • IFN-γ↑→IDO1↑→production↑ • KMO activity↑→QUIN/KYNA ratio↑	• NMDA receptors • Mitochondrial complex I	1.activationNMDA-R→Ca2+ internal flow↑→mPTPopen 2.inhibitionETC→ROS↑→mtDNAdamage 3.activationPARP→NAD+exhaustion	•Physiological concentration (≤100 nM): Neurotransmitter regulation •High concentrations: Excitotoxicity	AD Ischemic stroke	•Mitochondrial swelling→Cytochromecrelease→caspase-3Activation • Astrocyte mitochondrial fragmentation → ATP synthesis↓	(Beggiato et al., 2014; Zhao et al., 2019)
Kynurenic acid (KYNA)	source: •astrocytesKATsRegulation: • KMO inhibition → production ↑ inflammatory environment → KAT activity ↓	• NMDAreceptors • α7nAChRantagonists	1.Block NMDA-R→Excitotoxicity↓ 2.scavenging free radicals	•Neuroprotection •Antioxidant	Schizophrenia Huntington disease	• QUINToxicity not antagonized→Neuronal death ↑ •Glutamatergic signaling disorders	(Li et al., 2025)
Cross-regulatory mechanism	Imbalance in the microbiota-host metabolic axis	–	1. Inflammatory factors (IFN-γ/TNF-α) ↑ → IDO1 ↑ → KP shunt ↑ → Trp substrate ↓ 2. Indole-producing bacteria ↓ (F. prausnitzii/Lactobacillus) → IPA/I3A ↓ 3. Insufficient AhR/PXR activation → Barrier damage → Endotoxin translocation ↑ → Exacerbated inflammation	• Maintain Trp metabolic balance	IBD AD	IBD:stoolIPA↓→AhR↓→Barrier damage→LPS↑→IDO1↑→KP↑ • AD : IntestinalIPA↓→BBBPermeability↑→neuroinflammation↑→IDO1↑	(Lamas et al., 2016; Huang et al., 2023)

### 2.5 The serotonin pathway: bridging the gut and brain

While the KP is the major catabolic route, the serotonin pathway represents a critical anabolic branch of tryptophan metabolism with profound systemic influence. The synthesis of 5-HT is initiated by the rate-limiting enzyme tryptophan hydroxylase (TPH), which exists in two isoforms: TPH1, predominantly expressed in the gut enterochromaffin cells, and TPH2, primarily found in the central nervous system (CNS) (Imamdin and Van Der Vorst, 2023; Sinenko et al., 2023). This anatomical distinction underlies the concept of dual serotoninergic systems: over 90% of the body’s 5-HT is produced in the periphery (primarily in the gut), while CNS-derived 5-HT acts as a key neurotransmitter (Loh et al., 2024).

The gut microbiota is a pivotal regulator of peripheral 5-HT synthesis. Specific spore-forming microbes and metabolites from other commensals can directly stimulate colonic enterochromaffin cells to produce 5-HT (Loh et al., 2024). Gut-derived 5-HT cannot cross the blood-brain barrier but exerts extensive peripheral effects, including modulating gastrointestinal motility, platelet aggregation, and bone metabolism (Chang, 2024). Furthermore, through its actions on vagal afferents and immune cells, peripheral 5-HT plays a significant role in gut-brain axis communication (Chang, 2024).

Within the CNS, 5-HT is a quintessential neurotransmitter regulating mood, cognition, sleep, and appetite. Dysregulation of the central serotonin pathway is a cornerstone of neuropsychiatric disorders such as major depressive disorder, anxiety, and migraines (Zheng et al., 2025). The pathway is also intricately linked with the KP: inflammatory cytokines can shift tryptophan metabolism away from 5-HT synthesis and towards the KP, thereby reducing 5-HT bioavailability and contributing to inflammation-associated depressive symptoms. This crosstalk highlights the metabolic competition between the pathways and their collective impact on brain function and behavior.

## 3 Molecular mechanisms: metabolic switches and signaling hubs

The Try metabolic network plays a physiological protective role in maintaining homeostasis that transcends its pathological role. The metabolite-receptor interaction system formed by the co-evolution of microorganisms and their hosts builds a multi-organ defense system by precisely regulating epithelial integrity, neurovascular function, and energy metabolism balance (Chang, 2024). This section will systematically explain the core mechanism of tryptophan metabolites as endogenous protective mediators.

### 3.1 The AHR-IL-22 axis

This section elaborates on a key downstream axis of the AHR signaling pathway, introduced in the overview, which is central to maintaining intestinal homeostasis.

Indole derivatives produced by the gut microbiota, such as IPA, act as high-affinity ligands for the AHR. These ligands activate AHR signaling in intestinal epithelial cells and type 3 innate lymphoid cells (ILC3s), helping to maintain a dynamic intestinal barrier defense (Aoki et al., 2018). Direct transcriptional regulation of AHR induces enhanced expression of ZO-1 and mucins (MUC2), significantly improving physical barrier function and limiting pathogen and toxin translocation. More importantly, AHR activation by ILC3s triggers the secretion of interleukin-22(IL-22), which binds to the IL-22 receptor on epithelial cells, activates the STAT3 phosphorylation cascade, drives goblet cell proliferation and mucus layer thickening, and promotes the proliferation of Lgr5+ intestinal stem cells to accelerate epithelial damage repair (Hou et al., 2021; Ishihara et al., 2022). Clinical studies have confirmed that AHR-agonistic indole levels in the feces of patients with IBD are significantly positively correlated with serum IL-22 concentrations(*p* < 0.01), and that their deficiency is directly associated with increased intestinal permeability(elevated plasma lactoferrin levels) and worsening disease activity index(CDAI) (Wang et al., 2023). Concurrently, the AHR-IL-22 axis establishes an immune tolerance microenvironment at the mucosal interface by inhibiting RORγt-mediated Th17 cell differentiation and proinflammatory factor (IL-17, TNF-α) release, while inducing Foxp3+ regulatory Treg cell expansion (Cui et al., 2024). Therefore, the microbiota-tryptophan-AHR-IL-22-ZO1 pathway constitutes the core regulatory loop of intestinal homeostasis, and its dysregulation is a common basis for the pathogenesis of IBD.

### 3.2 Metabolic defense of neurovascular units

In the brain, tryptophan metabolites provide neuroprotection by regulating the neurovascular unit (NVU). For example, 3-HKA promotes vascular remodeling. Following cerebral ischemia injury, activated microglia and infiltrating macrophages exhibit upregulation of KYNU expression, which catalyzes the conversion of 3-HK to 3-HKA (Kang et al., 2024). 3-HKA, as an endogenous agonist of the metabolic glutamate receptor 4(mGluR4), binds to mGluR4 on the surface of astrocytes, activating the calcium ion/calmodulin kinase II(CaMKII)-cAMP response element-binding protein(CREB) signaling axis, promoting the transformation of astrocytes from the pro-inflammatory A1 phenotype(C3+) to the neuroprotective A2 phenotype(S100A10+) (Chen et al., 2025). A2-type astrocytes release vascular endothelial growth factor(VEGF), brain-derived neurotrophic factor(BDNF), and transforming growth factor-*β*(TGF-*β*), which directly stimulate the proliferation and migration of vascular endothelial cells, promote angiogenesis in the penumbra, and enhance the reconstruction of tight junctions in the blood-brain barrier (Wang et al., 2022; Li et al., 2025). In a permanent middle cerebral artery occlusion(pMCAO) model, intraventricular injection of 3-HKA reduced infarct volume by 38% ± 5%(*p* < 0.001), accompanied by increased vascular density and neurological recovery (Li et al., 2025).

Consistent with its well-established neuroprotective role (as overviewed in Part 1), KYNA antagonizes NMDAR-mediated Ca^2+^influx in ischemia, thereby supporting neuronal survival (Juhász et al., 2025). In the ischemia-reperfusion model, KYNA levels in the hippocampal CA1 region were positively correlated with neuronal survival(*r* = 0.72), while KATII gene knockout mice exhibited exacerbated excitatory neuronal death (Wang et al., 2022). In addition, KYNA activates AHR in astrocytes, inducing the expression of phase II detoxification enzymes such as quinone oxidoreductase (NQO1), which synergistically clears ischemia-related ROS storms (Ross and Siegel, 2021). Therefore, the signaling pathway mediated by 3-HKA activation of metabolic glutamate receptor 4(mGluR4) that promotes the conversion of astrocytes to the neuroprotective A2 phenotype and ultimately induces angiogenesis, and the neuroprotective effects provided by KYNA through antagonizing NMDAR and exerting antioxidant effects, synergistically constitute a dual neurovascular protective mechanism. This endogenous mechanism provides potential therapeutic targets for the repair of stroke and neurodegenerative diseases.

### 3.3 Microecological regulation of metabolic homeostasis

Intestinal microbiota-derived indole compounds IPA and IAA stimulate GLP-1 secretion through two main pathways—the gut-pancreas axis—to regulate insulin/glucagon and directly act on the liver and adipose tissue to inhibit hepatic gluconeogenesis, improve insulin sensitivity, and promote fat thermogenesis, thereby achieving fine regulation of systemic glucose homeostasis and energy metabolism balance.

In the regulation of the gut-pancreatic axis, IPA and IAA activate G protein-coupled receptor 119(GPR119) on the surface of enteroendocrine L cells, stimulating the secretion of glucagon-like peptide-1 (GLP-1) through the cAMP/PKA signaling pathway (Drucker, 2001; Meier et al., 2002a; Gromada et al., 2004). GLP-1 promotes insulin synthesis and release by pancreatic beta cells in a glucose concentration-dependent manner, while inhibiting glucagon secretion by alpha cells (Carlessi et al., 2017). In lean and obese rat models, a high-protein diet significantly reduced the area under the blood glucose curve (AUC) during the oral glucose tolerance test (OGTT) in obese rats. The increase in GLP-1 AUC and elevated fasting insulin levels indicate that the high-protein diet improved the rats’ tolerance to diabetes (Reimer and Russell, 2008). IPA directly inhibits the transcription of key rate-limiting enzymes in hepatic gluconeogenesis, phosphoenolpyruvate carboxylase kinase(PEPCK) and glucose-6-phosphatase (G6Pase), through the AHR-peroxisome proliferator-activated receptor gamma (PPAR*γ*) axis, thereby reducing endogenous glucose output (Untereiner et al., 2016). At the same time, AHR activation enhances tyrosine phosphorylation of insulin receptor substrate 1(IRS1), improving insulin signaling (Run et al., 2023). IAA activates the PXR, induces browning of white adipose tissue(WAT), upregulates uncoupling protein 1(UCP1) and genes related to mitochondrial biogenesis, and promotes thermogenic energy expenditure (Gong et al., 2024; Wang et al., 2025). Clinical studies have shown that serum IPA concentrations in patients with type 2 diabetes are significantly negatively correlated with the insulin resistance index (HOMA-IR)(*p*<-0.065), and low IPA levels independently predict the risk of developing diabetes (Ballanti et al., 2024).

## 4 Tryptophan metabolic network dysregulation and therapeutic targets: mechanisms and interventions in chronic diseases

Disruption of the tryptophan metabolic network plays a crucial role in the pathogenesis of chronic diseases, driving disease onset and progression by impairing immune function (Zhao et al., 2018), neuroprotection (Messaoud et al., 2019), and metabolic balance (Szelest et al., 2021; Stone and Williams, 2023). At the same time, the complexity and multifunctionality of the tryptophan metabolic network provide diverse intervention targets for the treatment of chronic diseases. This section will systematically analyze the molecular mechanisms of tryptophan metabolism disorders in autoimmune diseases and neurodegenerative diseases and discuss the challenges and opportunities in clinical practice from the perspective of mechanism discovery.

### 4.1 Metabolic imbalance in autoimmune diseases and neurodegenerative diseases

#### 4.1.1 Role of tryptophan metabolism in systemic lupus erythematosus

In systemic lupus erythematosus (SLE), an inflammatory shift toward the kynurenine pathway depletes tryptophan, limiting the substrate available for serotonin synthesis and contributing to the high prevalence of mood disorders in these patients (Zhu et al., 2020; Eryavuz Onmaz et al., 2023). QUIN-driven excitotoxicity contributes to neuronal injury in NPSLE and stroke, whereas interventions that shift KP flux toward KYNA may alleviate these outcomes (Andrabi et al., 2011). In addition, studies have shown that IDO1 and IDO2 mRNA expression are most significantly upregulated in the spleen, and AHR is a necessary condition for the induction of IDO1 and IDO2 expression by 2,3,7,8-tetrachlorodibenzodioxin(TCDD) (Vogel et al., 2008). AHR regulates Blimp-1 expression through Bach2, inhibiting the differentiation of B cells into plasma cells *in vitro* (Vaidyanathan et al., 2017). This indicates that weakening AHR signal activation will reduce the clearance of autoreactive B cells, and that the inactivation of the IDO2-mediated B cell tolerance mechanism is a core component of SLE immune dysregulation.

#### 4.1.2 Tryptophan metabolism in rheumatoid arthritis

Tryptophan metabolism disorder plays a crucial role in autoimmune diseases such as rheumatoid arthritis(RA). Recent clinical metabolomics studies have provided direct evidence for this: compared with healthy controls, the levels of multiple metabolites in the Tryptophan-KP in the serum of newly diagnosed RA patients were significantly downregulated, and the levels of these metabolites were negatively correlated with disease activity indices(DAS28) and autoantibodies(anti-CCP) (Wu et al., 2025). More importantly, this metabolic disorder is associated with key immune imbalances: decreased tryptophan and kynurenine levels are associated with increased Th17/Treg ratios and Tfh/Tfr ratios (Zheng et al., 2024). This indicates that IDO1-mediated downregulation of the Tryptophan-KP is a key driver of immune tolerance disruption in rheumatoid arthritis. Preclinical studies have shown that L-Kyn-activated AHR promotes the differentiation of immature CD4+ Th cells into regulatory Treg cell phenotypes, while inhibiting differentiation into Th17 cells that produce IL-17 (Mezrich et al., 2010; Liu et al., 2017). This provides a new theoretical basis for correcting immune imbalances in RA by regulating tryptophan metabolism.

#### 4.1.3 Tryptophan metabolism in stroke and neurodegenerative diseases

In stroke and age-related neurodegenerative diseases, disorders in the KP and Tryptophan metabolism have a critical impact on neuronal function and disease progression (Huang et al., 2023). In the pathological process of stroke, due to the lack of KMO expression in astrocytes, the main astrocytic product of tryptophan catabolism is KYNA (Guillemin et al., 2005). As a metabolite of tryptophan, KYNA exerts its neuroregulatory function by regulating multiple neurotransmitter systems. Studies have shown that low nanomolar concentrations of KYNA can inhibit glutamate release in the caudate nucleus region of the striatum and have a widespread effect on the extracellular levels of acetylcholine, GABA, and dopamine (Amori et al., 2009; Zmarowski et al., 2009; Wu et al., 2010; Majeed et al., 2021). *In vivo* experiments further confirmed that fluctuations in KYNA levels directly regulate the release dynamics of glutamine, acetylcholine, and dopamine.

#### 4.1.4 Impact of aging on tryptophan metabolism

There is also a causal relationship between age-related neurodegeneration and tryptophan metabolism disorders. In aged mice(24 months old), the levels of SIRT1 protein in the hippocampus were significantly reduced (Quintas et al., 2012). Tryptophan supplementation may reduce the mRNA levels of pro-inflammatory cytokine genes and enhance mitochondrial function by increasing the mRNA levels of mitochondrial transcription factor A, nuclear respiratory factor 1, mitochondrial transcription factor B1, AMPKα1, AMPKα2, Sirt1, and PGC1α mRNA levels, as well as the protein expression of phosphorylated AMPK, Sirt1, and PGC1α, thereby enhancing mitochondrial function (Liu et al., 2023). Mitochondria are the core hub of aging regulation, and strategies to enhance their function can slow down the aging process in multiple ways, providing key targets for anti-aging interventions.

### 4.2 Therapeutic interventions targeting the tryptophan metabolic network

The complexity and compensatory potential of the tryptophan metabolic network necessitate diverse therapeutic strategies. A critical lesson from clinical trials, such as the failure of the IDO1 inhibitor epacadostat (Garber, 2018). It is crucial to develop a biomarker approach that can be applied to different tissues in order to realize the full potential of tryptophan metabolism networks in precision medicine. This requires establishing a biomarker screening and validation system with high specificity, sensitivity, and wide applicability from the perspectives of multi omics data integration, disease mechanism analysis, and individualized treatment decision-making. The core of this system lies in integrating multi-level data from the genome, epigenome, transcriptome, proteome, and metabolome, combined with medical imaging omics and liquid biopsy information, using artificial intelligence and machine learning models to identify driving biological features closely related to clinical outcomes and treatment responses (Alagarswamy et al., 2024). On this basis, candidate biomarkers must undergo rigorous mechanism validation and clinical association testing to ensure that they accurately reflect the pathological and physiological status of specific tissues, such as the level of quinoline acid(QA) in neuroinflammation or IDO1 activity in the tumor microenvironment (Wang et al., 2024). Furthermore, these biomarkers can be used for dynamic monitoring of treatment response and guiding patient stratification, thereby providing support for personalized treatment decisions, such as selecting KMO inhibitors for patients with high KMO expression or supplementing specific probiotics for patients with intestinal microbiota derived indole metabolite deficiency (Catani et al., 2025). Despite facing challenges such as organizational heterogeneity and standardized detection, by promoting the deep integration of computational science and clinical practice, and conducting long-term longitudinal research verification, this multi omics biomarker based strategy will greatly promote cross tissue precision therapy for tryptophan metabolism networks.

#### 4.2.1 Enzyme inhibitors

Rational drug design targeting key enzymes has advanced, though not without challenges. The failure of the highly selective IDO1 inhibitor epacadostat in a phase III melanoma trial(ECHO-301) highlighted several hurdles: compensatory upregulation of TDO2/KMO via HIF-1α-mediated metabolic reprogramming, continuous AHR activation by alternative micro environmental ligands, and a lack of predictive biomarkers for patient selection (Long et al., 2019). This has contributed to the development of next-generation inhibitors, such as the allosteric IDO1 inhibitor LY3381916, which shows promise in blocking the substrate channel and overcoming conformational resistance, though further validation through clinical trials is required (Dorsey et al., 2018). Beyond IDO1/TDO2, inhibition of downstream branchpoint enzymes is also promising. For instance, the KMO inhibitor CHDI-340246 potently and dose-dependently modulates the KP in transgenic Huntington’s disease models, inhibiting the formation of 3-HK and QUIN while elevating neuroprotective KYNA levels in the brain (Kudo et al., 2024; Tufail et al., 2024).

#### 4.2.2 Metabolite supplementation and receptor modulation

Direct administration of key metabolites offers a complementary approach to restore metabolic balance. In ischemic stroke, supplementation with 3-hydroxykynurenine(3-HK) has demonstrated significant therapeutic potential. In a middle cerebral artery occlusion(MCAO) model, 3-HK treatment reduced infarct volume and promoted long-term neurological recovery (Wang et al., 2022). Its mechanism involves multi-dimensional neurovascular unit reconstruction: it enhances angiogenesis and blood-brain barrier integrity by activating the VEGF signaling pathway, while simultaneously driving the polarization of astrocytes from a neurotoxic A1 to a neuroprotective A2 phenotype by inhibiting NF-κB and activating STAT3 (Wang et al., 2022; Li et al., 2025). This exemplifies the potential of metabolite-based therapy to orchestrate complex repair processes.

#### 4.2.3 Microbiome-targeted therapies

Precision modulation of the gut microbiota represents a powerful strategy to influence host tryptophan metabolism. Engineered probiotics that overexpress tryptophanase(tnaA) can significantly increase the production of indole derivatives like IPA (Li et al., 2011). Furthermore, specific AHR-activating ligands derived from the microbiome or diet have shown efficacy in ameliorating colitis in models such as DSS, TNBS, and T-cell transfer (Monteleone et al., 2011; Zelante et al., 2013; Goettel et al., 2016; Islam et al., 2017). The beneficial effects of these compounds are associated with elevated IL-22 levels and are attenuated by IL-22 blockade or AHR antagonism, confirming the critical role of the microbiota-AHR-IL-22 axis in their mechanism of action (Zelante et al., 2013; Bai et al., 2019).

When considering dietary Tryptophan supplementation or high-Tryptophan diets as a therapeutic strategy, two key confounding factors are paramount. First, bioavailability is limited by competition at the intestinal transporter level; high levels of other LNAAs can significantly reduce Tryptophan uptake into circulation and across the blood-brain barrier, thereby limiting its availability for both host and microbial metabolism. Second, the source and matrix of the diet introduce confounding compounds. For example, high-protein diets may provide more Tryptophan but also increase competing LNAAs, while complex plant-based diets provide additional AHR ligands and fibers that independently modulate the microbial ecosystem and host immunity. Therefore, a holistic view of the dietary composition, rather than focusing on a single nutrient, is essential for predicting intervention outcomes.

#### 4.2.4 Natural products with multi-target activity

Natural products often exert synergistic effects through multi-target regulation. Shikimic acid(SA), for example, inhibits Staphylococcus aureus biofilm formation by modulating sarA and agrA transcription and exerts cardioprotective effects via antioxidant and anti-inflammatory mechanisms (Bai et al., 2019; Alwaili et al., 2025). More notably, artemisinin and its derivatives have proven to be effective anticancer agents. They activate the mitochondrial ROS-JNK pathway to induce apoptosis while concurrently downregulating IDO1 expression. Artemisinin dimers can target heme-dependent IDO1 for degradation, reducing tumor KYN levels by approximately 75% and synergizing with anti-PD-1 therapy (Stockwin et al., 2009; Berdelle et al., 2011; Xia et al., 2025). These findings highlight the potential of natural products in multi-target cancer and infection treatments.

## 5 Challenges and future perspectives

Future challenges in applying insights from tryptophan metabolism to clinical practice mainly involve technical and biological hurdles. One challenge is the limited ability to detect metabolites in specific tissues over time, especially for metabolites like kynurenine. To address this, combining spatial metabolomics with single-cell transcriptomics is necessary to better understand metabolic changes at a cellular level.

Another challenge is the complexity of host-microbiome interactions in metabolism. Current models do not fully integrate microbial and host metabolic data. Developing better models that combine metabolomic, microbiomic, and enzymatic data will be crucial for understanding how metabolic changes affect disease and treatment responses.

Therapeutic strategies are also limited by metabolic redundancy and individual variability. Single-target therapies may not be effective due to compensatory mechanisms. Dual-target inhibitors and combination therapies are needed to address this complexity. Additionally, variations in microbiome composition affect treatment outcomes, so precision medicine must account for these differences.

Emerging technologies offer solutions to these challenges. Engineering metabolites and using probiotics to enhance beneficial microbial metabolites could lead to new treatments. AI-driven models can help identify new therapeutic targets and improve drug development. (Xia et al., 2025a; Xia et al., 2025b; Xia et al., 2025c; Xia et al., 2025d)

Looking ahead, research should focus on validating tissue-specific biomarkers and integrating spatial metabolomics with artificial intelligence technology to refine treatment strategies (Chen et al., 2024). Understanding dietary factors and tryptophan bioavailability will also be important for developing personalized treatments. These efforts will help advance precision medicine, particularly in cancer, neurology, and immunology.

## References

[B1] Adams S. Braidy N. Bessede A. Brew B. J. Grant R. Teo C. . (2012). The kynurenine pathway in brain tumor pathogenesis. Cancer Res. 72, 5649–5657. doi: 10.1158/0008-5472.CAN-12-0549, PMID: 23144293

[B2] Alagarswamy K. Shi W. Boini A. Messaoudi N. Grasso V. Cattabiani T. . (2024). Should AI-powered whole-genome sequencing be used routinely for personalized decision support in surgical oncology-A scoping review. BioMedInformatics 4, 1757–1772. doi: 10.3390/biomedinformatics4030096

[B3] Alwaili M. A. Abu-Almakarem A. S. El-Said K. S. Eid T. M. Mobasher M. A. Alsabban A. H. . (2025). Shikimic acid protects against doxorubicin-induced cardiotoxicity in rats. Sci. Rep. 15, 8126. doi: 10.1038/s41598-025-90549-4, PMID: 40057537 PMC11890735

[B4] Amori L. Guidetti P. Pellicciari R. Kajii Y. Schwarcz R. (2009). On the relationship between the two branches of the kynurenine pathway in the rat brain *in vivo*. J. Neurochem. 109, 316–325. doi: 10.1111/j.1471-4159.2009.05893.x, PMID: 19226371 PMC3666345

[B5] Andrabi S. A. Kang H. C. Haince J. F. Lee Y. I. Zhang J. Chi Z. . (2011). Iduna protects the brain from glutamate excitotoxicity and stroke by interfering with poly(ADP-ribose) polymer-induced cell death. Nat. Med. 17, 692–699. doi: 10.1038/nm.2387, PMID: 21602803 PMC3709257

[B6] Aoki R. Aoki-Yoshida A. Suzuki C. Takayama Y. (2018). Indole-3-Pyruvic Acid, an aryl hydrocarbon receptor activator, suppresses experimental colitis in mice. J. Immunol. 201, 3683–3693. doi: 10.4049/jimmunol.1701734, PMID: 30429284

[B7] Badawy A. A. (2017). Kynurenine pathway of tryptophan metabolism: Regulatory and functional aspects. Int. J. Tryptophan Res. 10, 1178646917691938. doi: 10.1177/1178646917691938, PMID: 28469468 PMC5398323

[B8] Bai J. R. Zhong K. Wu Y. P. Grosu E. Gao H. (2019). Antibiofilm activity of shikimic acid against Staphylococcus aureus. Food Ctrl 95, 327–333. doi: 10.1016/j.foodcont.2018.08.020

[B9] Ballanti M. Antonetti L. Mavilio M. Casagrande V. Moscatelli A. Pietrucci D. . (2024). Decreased circulating IPA levels identify subjects with metabolic comorbidities: A multi-omics study. Pharmacol. Res. 204, 107207. doi: 10.1016/j.phrs.2024.107207, PMID: 38734193

[B10] Bansal Y. Singh R. Parhar I. Kuhad A. Soga T. (2019). Quinolinic acid and nuclear factor erythroid 2-related factor 2 in depression: role in neuroprogression. Front. Pharmacol. 10, 452. doi: 10.3389/fphar.2019.00452, PMID: 31164818 PMC6536572

[B11] Basak U. Sarkar T. Mukherjee S. Chakraborty S. Dutta A. Dutta S. . (2023). Tumor-associated macrophages: An effective player of the tumor microenvironment. Front. Immunol. 14, 1295257. doi: 10.3389/fimmu.2023.1295257, PMID: 38035101 PMC10687432

[B12] Beaumont V. Mrzljak L. Dijkman U. Freije R. Heins M. Rombouts F. . (2016). The novel KMO inhibitor CHDI-340246 leads to a restoration of electrophysiological alterations in mouse models of Huntington’s disease. Exp. Neurol. 282, 99–118. doi: 10.1016/j.expneurol.2016.05.005, PMID: 27163548

[B13] Beggiato S. Tanganelli S. Fuxe K. Antonelli T. Schwarcz R. Ferraro L. (2014). Endogenous kynurenic acid regulates extracellular GABA levels in the rat prefrontal cortex. Neuropharmacology 82, 11–18. doi: 10.1016/j.neuropharm.2014.02.019, PMID: 24607890

[B14] Bellucci M. Pompa A. De Marcos Lousa C. Panfili E. Orecchini E. Maricchiolo E. . (2021). Human indoleamine 2,3-dioxygenase 1(IDO1) expressed in plant cells induces kynurenine production. Int. J. Mol. Sci. 22, 5102. doi: 10.3390/ijms22105102, PMID: 34065885 PMC8151846

[B15] Berdelle N. Nikolova T. Quiros S. Efferth T. Kaina B. (2011). Artesunate induces oxidative DNA damage, sustained DNA double-strand breaks, and the ATM/ATR damage response in cancer cells. Mol. Cancer Ther. 10, 2224–2233. doi: 10.1158/1535-7163.MCT-11-0534, PMID: 21998290

[B16] Bröer A. Rahimi F. Bröer S. (2016). Deletion of amino acid transporter ASCT2(SLC1A5) reveals an essential role for transporters SNAT1(SLC38A1) and SNAT2(SLC38A2) to sustain glutaminolysis in cancer cells. J. Biol. Chem. 291, 13194–13205. doi: 10.1074/jbc.M115.700534, PMID: 27129276 PMC4933233

[B17] Brundin L. Sellgren C. M. Lim C. K. Grit J. Pålsson E. Landén M. . (2016). An enzyme in the kynurenine pathway that governs vulnerability to suicidal behavior by regulating excitotoxicity and neuroinflammation. Transl. Psychiatry 6, e865. doi: 10.1038/tp.2016.133, PMID: 27483383 PMC5022080

[B18] Carlessi R. Chen Y. Rowlands J. Cruzat V. F. Keane K. N. Egan J. . (2017). GLP-1 receptor signalling promotes β-cell glucose metabolism via mTOR-dependent HIF-1α activation. Sci. Rep. 7, 2661. doi: 10.1038/s41598-017-02838-2, PMID: 28572610 PMC5454020

[B19] Catani G. Morchón-Araujo D. Mirallas O. Sánchez-Pérez V. Nuciforo P. Villacampa G. . (2025). Optimizing early-phase immunotherapy trials: the role of biomarker enrichment strategies. Front. Immunol. 16. doi: 10.3389/fimmu.2025.1664443, PMID: 41200168 PMC12586187

[B20] Cellini B. Zelante T. Dindo M. Bellet M. M. Renga G. Romani L. . (2020). Pyridoxal 5’-phosphate-dependent enzymes at the crossroads of host-microbe tryptophan metabolism. Int. J. Mol. Sci. 21, 5823. doi: 10.3390/ijms21165823, PMID: 32823705 PMC7461572

[B21] Chang P. V. (2024). Microbial metabolite-receptor interactions in the gut microbiome. Curr. Opin. Chem. Biol. 83, 102539. doi: 10.1016/j.cbpa.2024.102539, PMID: 39461049 PMC11588511

[B22] Chen C. J. Kimble B. Van Aggelen A. Fischer S. Flanagan C. Gillett A. . (2024). Preliminary analyses of tryptophan, kynurenine, and the kynurenine: Tryptophan ratio in plasma, as potential biomarkers for systemic chlamydial infections in koalas. PloS One 19, e0314945. doi: 10.1371/journal.pone.0314945, PMID: 39700217 PMC11658483

[B23] Chen J. M. Shi G. Yu L. L. Shan W. Sun J. Y. Guo A. C. . (2025). 3-HKA promotes vascular remodeling after stroke by modulating the activation of A1/A2 reactive astrocytes. Adv. Sci. 12, e2412667. doi: 10.1002/advs.202412667, PMID: 39854137 PMC11923925

[B24] Cormerais Y. Massard P. A. Vucetic M. Giuliano S. Tambutté É. Durivault J. . (2018). The glutamine transporter ASCT2(SLC1A5) promotes tumor growth independently of the amino acid transporter LAT1(SLC7A5). J. Biol. Chem. 293, 2877–2887. doi: 10.1074/jbc.RA117.001342, PMID: 29326164 PMC5827425

[B25] Cui H. Wang N. Li H. Bian Y. Wen W. Kong X. . (2024). The dynamic shifts of IL-10-producing Th17 and IL-17-producing Treg in health and disease: A crosstalk between ancient “Yin-Yang” theory and modern immunology. Cell Commun. Signal 22, 99. doi: 10.1186/s12964-024-01505-0, PMID: 38317142 PMC10845554

[B26] Dhuguru J. Dellinger R. W. Migaud M. E. (2023). Defining NAD(P)(H) catabolism. Nutrients 15, 3064. doi: 10.3390/nu15133064, PMID: 37447389 PMC10346783

[B27] Dorsey F. C. Benhadji K. A. Sams L. L. Young D. A. Schindler J. F. Huss K. L. . (2018). Identification and characterization of the IDO1 inhibitor LY3381916. Cancer Res. 78, 5245. doi: 10.1158/1538-7445.AM2018-5245

[B28] Drucker D. J. (2001). Development of glucagon-like peptide-1-based pharmaceuticals as therapeutic agents for the treatment of diabetes. Curr. Pharm. Des. 7, 1399–1412. doi: 10.2174/1381612013397401, PMID: 11472275

[B29] Dudzinska E. Szymona K. Kloc R. Gil-Kulik P. Kocki T. Swistowska M. . (2019). Increased expression of kynurenine aminotransferases mRNA in lymphocytes of patients with inflammatory bowel disease. Ther Adv. Gastroenterol. 12. doi: 10.1177/1756284819881304, PMID: 31666808 PMC6801885

[B30] El-Azaz J. Moore B. Takeda-Kimura Y. Yokoyama R. Wijesingha Ahchige M. Chen X. . (2023). Coordinated regulation of the entry and exit steps of aromatic amino acid biosynthesis supports the dual lignin pathway in grasses. Nat. Commun. 14, 7242. doi: 10.1038/s41467-023-42587-7, PMID: 37945591 PMC10636026

[B31] Eryavuz Onmaz D. Tezcan D. Yilmaz S. Onmaz M. Unlu A. (2023). Altered kynurenine pathway metabolism and association with disease activity in patients with systemic lupus. Amino Acids 55, 1937–1947. doi: 10.1007/s00726-023-03353-7, PMID: 37925676

[B32] Fargher E. Keatinge M. Pearce O. Piepponen P. Panula P. van Eeden F. J.M. . (2025). A zebrafish model of acmsd deficiency does not support a prominent role for ACMSD in Parkinson’s disease. NPJ Parkinsons Dis. 11, 118. doi: 10.1038/s41531-025-00940-1, PMID: 40346140 PMC12064770

[B33] Fiedorowicz M. Choragiewicz T. Turski W. Kocki T. Nowakowska D. Wertejuk K. . (2021). Tryptophan pathway abnormalities in a murine model of hereditary glaucoma. Int. J. Mol. Sci. 22, 1039. doi: 10.3390/ijms22031039, PMID: 33494373 PMC7865582

[B34] Garber K. (2018). A new cancer immunotherapy suffers a setback. Science 360, 588. doi: 10.1126/science.360.6389.588, PMID: 29748264

[B35] Garg P. Malhotra J. Kulkarni P. Horne D. Salgia R. Singhal S. S. (2024). Emerging therapeutic strategies to overcome drug resistance in cancer cells. Cancers 16, 2478. doi: 10.3390/cancers16132478, PMID: 39001539 PMC11240358

[B36] Goettel J. A. Gandhi R. Kenison J. E. Yeste A. Murugaiyan G. Sambanthamoorthy S. . (2016). AHR activation is protective against colitis driven by T cells in humanized mice. Cell Rep. 17, 1318–1329. doi: 10.1016/j.celrep.2016.09.082, PMID: 27783946 PMC5106873

[B37] Gong D. Lei J. He X. Hao J. Zhang F. Huang X. . (2024). Keys to the switch of fat burning: Stimuli that trigger the uncoupling protein 1(UCP1) activation in adipose tissue. Lipids Health Dis. 23, 322. doi: 10.1186/s12944-024-02300-z, PMID: 39342273 PMC11439242

[B38] Gromada J. Brock B. Schmitz O. Rorsman P. (2004). Glucagon-like peptide-1: Regulation of insulin secretion and therapeutic potential. Basic Clin. Pharmacol. Toxicol. 95, 252–262. doi: 10.1111/j.1742-7843.2004.t01-1-pto950502.x, PMID: 15569269

[B39] Guangzhao L. Xin W. Miaoqing W. Wenjuan M. Ranyi L. Zhizhong P. . (2025). IDO1 inhibitor enhances the effectiveness of PD-1 blockade in microsatellite stable colorectal cancer by promoting macrophage pro-inflammatory phenotype polarization. Cancer Immunol. Immunother. 74, 71. doi: 10.1007/s00262-024-03925-w, PMID: 39751692 PMC11699167

[B40] Guillemin G. J. Brew B. J. Noonan C. E. Takikawa O. Cullen K. M. (2005). Indoleamine 2,3-dioxygenase and quinolinic acid immunoreactivity in Alzheimer’s disease hippocampus. Neuropathol. Appl. Neurobiol. 31, 395–404. doi: 10.1111/j.1365-2990.2005.00655.x, PMID: 16008823

[B41] Ha E. Antonios J. P. Soto H. Prins R. M. Yang I. Kasahara N. . (2014). Chronic inflammation drives glioma growth: Cellular and molecular factors responsible for an immunosuppressive microenvironment. Front. Immunol. 5. doi: 10.4103/2347-8659.139717

[B42] Hou Q. Huang J. Ayansola H. Masatoshi H. Zhang B. (2021). Intestinal stem cells and immune cell relationships: Potential therapeutic targets for inflammatory bowel diseases. Front. Immunol. 11, 623691. doi: 10.3389/fimmu.2020.623691, PMID: 33584726 PMC7874163

[B43] Hsu C. N. Lin I.-C. Yu H. R. Huang L. T. Tiao M. M. Tain Y. L. (2020). Maternal tryptophan supplementation protects adult rat offspring against hypertension programmed by maternal chronic kidney disease: Implication of tryptophan-metabolizing microbiome and aryl hydrocarbon receptor. Int. J. Mol. Sci. 21, 4552. doi: 10.3390/ijms21124552, PMID: 32604820 PMC7349830

[B44] Hu W. Garrison C. Prasad R. Boulton M. E. Grant M. B. (2025). Indole metabolism and its role in diabetic macrovascular and microvascular complications. Am. Heart J. Plus Cardiol. Res. Pract. 53, 100532. doi: 10.1016/j.ahjo.2025.100532, PMID: 40230659 PMC11995707

[B45] Huang K. Han Y. Chen Y. Shen H. Zeng S. Cai C. (2025). Tumor metabolic regulators: Key drivers of metabolic reprogramming and promising targets in cancer therapy. Mol. Cancer 24, 7. doi: 10.1186/s12943-024-02205-6, PMID: 39789606 PMC11716519

[B46] Huang Z. Xie L. Huang L. (2023). Regulation of host immune responses by Lactobacillus through aryl hydrocarbon receptors. Med. Microecol 16, 100081. doi: 10.1016/j.medmic.2023.100081

[B47] Huang Y. Zhao M. Chen X. Zhang R. Le A. Hong M. . (2023). Tryptophan metabolism in central nervous system diseases: Pathophysiology and potential therapeutic strategies. Aging Dis. 14, 858–878. doi: 10.14336/AD.2022.0916, PMID: 37191427 PMC10187711

[B48] Imamdin A. Van Der Vorst E. P. C. (2023). Exploring the role of serotonin as an immune modulatory component in cardiovascular diseases. Int. J. Mol. Sci. 24, 1549. doi: 10.3390/ijms24021549, PMID: 36675065 PMC9861641

[B49] Ishihara Y. Kado S. Y. Bein K. J. He Y. Pouraryan A. A. Urban A. . (2022). Aryl hydrocarbon receptor signaling synergizes with TLR/NF-κB-signaling for induction of IL-22 through canonical and non-canonical. AHR pathways. Front. Toxicol. 3, 787360. doi: 10.3389/ftox.2021.787360, PMID: 35295139 PMC8915841

[B50] Islam J. Sato S. Watanabe K. Watanabe T. Ardiansyah Hirahara K. . (2017). Dietary tryptophan alleviates dextran sodium sulfate-induced colitis through aryl hydrocarbon receptor in mice. J. Nutr. Biochem. 42, 43–50. doi: 10.1016/j.jnutbio.2016.12.019, PMID: 28113104

[B51] Jamshed L. Debnath A. Jamshed S. Wish J. V. Raine J. C. Tomy G. T. . (2022). An emerging cross-species marker for organismal health: Tryptophan-kynurenine pathway. Int. J. Mol. Sci. 23, 6300. doi: 10.3390/ijms23116300, PMID: 35682980 PMC9181223

[B52] Jovanovic F. Candido K. D. Knezevic N. N. (2020). The role of the kynurenine signaling pathway in different chronic pain conditions and potential use of therapeutic agents. Int. J. Mol. Sci. 21, 6045. doi: 10.3390/ijms21176045, PMID: 32842609 PMC7503462

[B53] Juhász L. Spisák K. Szolnoki B. Z. Nászai A. Szabó Á Rutai A. . (2025). The power struggle: Kynurenine pathway enzyme knockouts and brain mitochondrial respiration. J. Neurochem. 169, e70075. doi: 10.1111/jnc.70075, PMID: 40317489 PMC12048769

[B54] Kang C. Sang Q. Liu D. Li R. Wang C. Guo F. . (2024). Polyphyllin I alleviates neuroinflammation after cerebral ischemia-reperfusion injury via facilitating autophagy-mediated M2 microglial polarization. Mol. Med. 30, 59. doi: 10.1186/s10020-024-00828-5, PMID: 38745316 PMC11094947

[B55] Kang I. Theodoropoulos G. Wangpaichitr M. (2025). Targeting the kynurenine pathway: another therapeutic opportunity in the metabolic crosstalk between cancer and immune cells. Front. Oncol. 14, 1524651. doi: 10.3389/fonc.2024.1524651, PMID: 39911818 PMC11794083

[B56] Kaur K. Celis A. P. Jewett A. (2025). Natural killer cell-secreted IFN-γ and TNF-α mediated differentiation in lung stem-like tumors, leading to the susceptibility of the tumors to chemotherapeutic drugs. Cells 14, 90. doi: 10.3390/cells14020090, PMID: 39851518 PMC11763808

[B57] Krishnamurthy H. K. Pereira M. Rajavelu I. Jayaraman V. Krishna K. Wang T. . (2024). Oxidative stress: fundamentals and advances in quantification techniques. Front. Chem. 12, 1470458. doi: 10.3389/fchem.2024.1470458, PMID: 39435263 PMC11491411

[B58] Krupa M. M. Pienkowski T. Tankiewicz-Kwedlo A. Lyson T. (2025). Targeting the kynurenine pathway in gliomas: Insights into pathogenesis, therapeutic targets, and clinical advances. Biochim. Biophys. Acta (BBA) - Rev. Cancer 1880, 189343. doi: 10.1016/j.bbcan.2025.189343, PMID: 40345262

[B59] Kudo K. Greer Y. E. Yoshida T. Ogiwara H. Cho H. Shergalis A. . (2024). Dual-inhibition of NAMPT and PAK4 induces anti-tumor effects in 3D-spheroids model of platinum-resistant ovarian cancer. Cancer Gene Ther. 31, 721–735. doi: 10.1038/s41417-024-00748-w, PMID: 38424218 PMC11101335

[B60] Lamas B. Richard M. L. Leducq V. Pham H. P. Michel M. L. Da Costa G. . (2016). CARD9 impacts colitis by altering gut microbiota metabolism of tryptophan into aryl hydrocarbon receptor ligands. Nat. Med. 22, 598–605. doi: 10.1038/nm.4102, PMID: 27158904 PMC5087285

[B61] Li Q. de Oliveira Formiga R. Puchois V. Creusot L. Ahmad A. H. Amouyal S. . (2025). Microbial metabolite indole-3-propionic acid drives mitochondrial respiration in CD4+ T cells to confer protection against intestinal inflammation. Nat. Metab. 7 (12), 2510–2530. doi: 10.1038/s42255-025-01396-6, PMID: 41120706 PMC12727523

[B62] Li Y. Innocentin S. Withers D. R. Roberts N. A. Gallagher A. R. Grigorieva E. F. . (2011). Exogenous stimuli maintain intraepithelial lymphocytes via aryl hydrocarbon receptor activation. Cell 147, 629–640. doi: 10.1016/j.cell.2011.09.025, PMID: 21999944

[B63] Li B. Liang X. Li Y. Wang R. Wei Y. Liu Q. . (2025). Tryptophan catabolites from microbiota ameliorate immune-mediated hepatitis through activating aryl hydrocarbon receptor of T cells. Gut Microbes 17, 2557979. doi: 10.1080/19490976.2025.2557979, PMID: 40995824 PMC12477874

[B64] Li W. Liu E. Zhou Y. Liao Z. Wang D. (2025). Therapeutic potential of natural products in ischemic stroke: Targeting angiogenesis. Front. Pharmacol. 16, 1579172. doi: 10.3389/fphar.2025.1579172, PMID: 40606624 PMC12213924

[B65] Liang H. Li T. Fang X. Liu Z. Song Q. Wang X. . (2021). IDO1/TDO dual inhibitor RY103 targets Kyn-.AHR pathway and exhibits preclinical efficacy on pancreatic cancer. Cancer Lett. 522, 32–43. doi: 10.1016/j.canlet.2021.09.012, PMID: 34520819

[B66] Liang D. Zhuo Y. Guo Z. He L. Wang X. He Y. . (2020). SIRT1/PGC-1 pathway activation triggers autophagy/mitophagy and attenuates oxidative damage in intestinal epithelial cells. Biochimie 170, 10–20. doi: 10.1016/j.biochi.2019.12.001, PMID: 31830513

[B67] Liu X. Hu H. Fan H. Zuo D. Shou Z. Liao Y. . (2017). The role of STAT3 and. AHR in the differentiation of CD4+ T cells into Th17 and Treg cells. Medicine 96, e6615. doi: 10.1097/MD.0000000000006615, PMID: 28445259 PMC5413224

[B68] Liu G. Sun W. Wang F. Jia G. Zhao H. Chen X. . (2023). Dietary tryptophan supplementation enhances mitochondrial function and reduces pyroptosis in the spleen and thymus of piglets after lipopolysaccharide challenge. Animal 17, 100714. doi: 10.1016/j.animal.2023.100714, PMID: 36764015

[B69] Liu H. Wang S. Wang J. Guo X. Song Y. Fu K. . (2025). Energy metabolism in health and diseases. Signal Transduct Tgt Ther. 10, 69. doi: 10.1038/s41392-025-02141-x, PMID: 39966374 PMC11836267

[B70] Loh J. S. Mak W. Q. Tan L. K. S. Ng C. X. Chan H. H. Yeow S. H. . (2024). Microbiota-gut-brain axis and its therapeutic applications in neurodegenerative diseases. Signal Transduct Tgt Ther. 9, 1. doi: 10.1038/s41392-024-01743-1, PMID: 38360862 PMC10869798

[B71] Long G. V. Dummer R. Hamid O. Gajewski T. F. Caglevic C. Dalle S. . (2019). Epacadostat plus pembrolizumab versus placebo plus pembrolizumab in patients with unresectable or metastatic melanoma(ECHO-301/KEYNOTE-252): A phase 3, randomised, double-blind study. Lancet Oncol. 20, 1083–1097. doi: 10.1016/S1470-2045(19)30274-8, PMID: 31221619

[B72] Madella A. M. Van Bergenhenegouwen J. Garssen J. Masereeuw R. Overbeek S. A. (2022). Microbial-derived tryptophan catabolites, kidney disease and gut inflammation. Toxins 14, 645. doi: 10.3390/toxins14090645, PMID: 36136583 PMC9505404

[B73] Madreiter-Sokolowski C. T. Thomas C. Ristow M. (2020). Interrelation between ROS and Ca2+ in aging and age-related diseases. Redox Biol. 36, 101678. doi: 10.1016/j.redox.2020.101678, PMID: 32810740 PMC7451758

[B74] Majeed Y. Halabi N. Madani A. Y. Engelke R. Bhagwat A. M. Abdesselem H. . (2021). SIRT1 promotes lipid metabolism and mitochondrial biogenesis in adipocytes and coordinates adipogenesis by targeting key enzymatic pathways. Sci. Rep. 11, 8177. doi: 10.1038/s41598-021-87759-x, PMID: 33854178 PMC8046990

[B75] Mandarano M. Orecchini E. Bellezza G. Vannucci J. Ludovini V. Baglivo S. . (2021). Kynurenine/tryptophan ratio as a potential blood-based biomarker in non-small cell lung cancer. Int. J. Mol. Sci. 22, 4403. doi: 10.3390/ijms22094403, PMID: 33922388 PMC8122814

[B76] Meier J. J. Nauck M. A. Schmidt W. E. Gallwitz B. (2002a). Gastric inhibitory polypeptide: The neglected incretin revisited. Regul. Pept. 107, 1–13. doi: 10.1016/S0167-0115(02)00039-3, PMID: 12137960

[B77] Messaoud A. Mensi R. Douki W. Neffati F. Najjar M. F. Gobbi G. . (2019). Reduced peripheral availability of tryptophan and increased activation of the kynurenine pathway and cortisol correlate with major depression and suicide. World J. Biol. Psychiatry 20, 703–711. doi: 10.1080/15622975.2018.1468031, PMID: 29683396

[B78] Mezrich J. D. Fechner J. H. Zhang X. Johnson B. P. Burlingham W. J. Bradfield C. A. (2010). An interaction between kynurenine and the aryl hydrocarbon receptor can generate regulatory T cells. J. Immunol. 185, 3190–3198. doi: 10.4049/jimmunol.0903670, PMID: 20720200 PMC2952546

[B79] Moffett J. R. Arun P. Puthillathu N. Vengilote R. Ives J. A. Badawy A. A.-B. . (2020). Quinolinate as a marker for kynurenine metabolite formation and the unresolved question of NAD+ synthesis during inflammation and infection. Front. Immunol. 11, 31. doi: 10.3389/fimmu.2020.00031, PMID: 32153556 PMC7047773

[B80] Moffett J. R. Espey M. G. Gaudet S. J. Namboodiri M. A. (1993). Antibodies to quinolinic acid reveal localization in select immune cells rather than neurons or astroglia. Brain Res. 623, 337–340. doi: 10.1016/0006-8993(93)91450-7, PMID: 8221118

[B81] Mondanelli G. Coletti A. Greco F. A. Pallotta M. T. Orabona C. Iacono A. . (2020). Positive allosteric modulation of indoleamine 2,3-dioxygenase 1 restrains neuroinflammation. Proc. Natl. Acad. Sci. U.S.A. 117, 3848–3857. doi: 10.1073/pnas.1918215117, PMID: 32024760 PMC7035626

[B82] Monteleone I. Rizzo A. Sarra M. Sica G. Sileri P. Biancone L. . (2011). Aryl hydrocarbon receptor-induced signals up-regulate IL-22 production and inhibit inflammation in the gastrointestinal tract. Gastroenterology 141, 237–248.e1. doi: 10.1053/j.gastro.2011.04.007, PMID: 21600206

[B83] Muneer A. (2020). Kynurenine pathway of tryptophan metabolism in neuropsychiatric disorders: Pathophysiologic and therapeutic considerations. Clin. Psychopharmacol. Neurosci. 18, 507–526. doi: 10.9758/cpn.2020.18.4.507, PMID: 33124585 PMC7609208

[B84] Natividad J. M. Agus A. Planchais J. Lamas B. Jarry A. C. Martin R. . (2018). Impaired aryl hydrocarbon receptor ligand production by the gut microbiota is a key factor in metabolic syndrome. Cell Metab. 28, 737–749.e4. doi: 10.1016/j.cmet.2018.07.001, PMID: 30057068

[B85] Niu B. Pan T. Xiao Y. Wang H. Zhu J. Tian F. . (2025). The therapeutic potential of dietary intervention: based on the mechanism of a tryptophan derivative-indole propionic acid on metabolic disorders. Crit. Rev. Food Sci. Nutr. 65, 1–20. doi: 10.1080/10408398.2023.2299744, PMID: 38189263

[B86] Pant K. Venugopal S. K. Lorenzo Pisarello M. J. Gradilone S. A. (2023). The role of gut microbiome-derived short-chain fatty acid butyrate in hepatobiliary diseases. Am. J. Pathol. 193, 1455–1467. doi: 10.1016/j.ajpath.2023.06.007, PMID: 37422149 PMC10548274

[B87] Peng R. Song C. Gou S. Liu Y. Wang Y. Ma C. . (2024). Gut Clostridium sporogenes-derived indole propionic acid suppresses osteoclast formation by activating pregnane X receptor. Pharmacol. Res. 202, 107121. doi: 10.1016/j.phrs.2024.107121, PMID: 38431091

[B88] Platten M. Friedrich M. Wainwright D. A. Panitz V. Opitz C. A. (2021). Tryptophan metabolism in brain tumors-IDO and beyond. Curr. Opin. Immunol. 70, 57–66. doi: 10.1016/j.coi.2021.03.005, PMID: 33813026 PMC8373719

[B89] Qu S. Gao Y. Ma J. Yan Q. (2023). Microbiota-derived short-chain fatty acids functions in the biology of B lymphocytes: From differentiation to antibody formation. BioMed. Pharmacother. 168, 115773. doi: 10.1016/j.biopha.2023.115773, PMID: 39491858

[B90] Quintas A. De Solís A. J. Díez-Guerra F. J. Carrascosa J. M. Bogónez E. (2012). Age-associated decrease of SIRT1 expression in rat hippocampus: Prevention by late onset caloric restriction. Exp. Gerontol 47, 198–201. doi: 10.1016/j.exger.2011.11.010, PMID: 22143179

[B91] Rakshe P. S. Dutta B. J. Chib S. Maurya N. Singh S. (2024). Unveiling the interplay of AMPK/SIRT1/PGC-1α axis in brain health: Promising targets against aging and NDDs. Ageing Res. Rev. 96, 102255. doi: 10.1016/j.arr.2024.102255, PMID: 38490497

[B92] Reimer R. A. Russell J. C. (2008). Glucose tolerance, lipids, and GLP-1 secretion in JCR: LA-cprats fed a high protein fiber diet. Obesity 16, 40–46. doi: 10.1038/oby.2007.16, PMID: 18223610 PMC3827014

[B93] Riess C. Schneider B. Kehnscherper H. Gesche J. Irmscher N. Shokraie F. . (2020). Activation of the kynurenine pathway in human Malignancies can be suppressed by the cyclin-dependent kinase inhibitor dinaciclib. Front. Immunol. 11, 115. doi: 10.3389/fimmu.2020.00055, PMID: 32117235 PMC7034242

[B94] Roager H. M. Licht T. R. (2018). Microbial tryptophan catabolites in health and disease. Nat. Commun. 9, 3294. doi: 10.1038/s41467-018-05470-4, PMID: 30120222 PMC6098093

[B95] Rocha G. Francés-Gómez C. Megías J. Muñoz-Hidalgo L. Casanova P. Haro-Estevez J. F. . (2025). Isocitrate dehydrogenase-wildtype glioma adapts toward mutant phenotypes and enhanced therapy sensitivity under D-2-hydroxyglutarate exposure. Biomedicines. 13, 1584. doi: 10.3390/biomedicines13071584, PMID: 40722659 PMC12292639

[B96] Ross D. Siegel D. (2021). The diverse functionality of NQO1 and its roles in redox control. Redox Biol. 41, 101950. doi: 10.1016/j.redox.2021.101950, PMID: 33774477 PMC8027776

[B97] Roth W. Zadeh K. Vekariya R. Ge Y. Mohamadzadeh M. (2021). Tryptophan metabolism and gut-brain homeostasis. Int. J. Mol. Sci. 22, 2973. doi: 10.3390/ijms22062973, PMID: 33804088 PMC8000752

[B98] Run L. Tian Z. Xu L. Du J. Li N. Wang Q. . (2023). Knockdown of IL4I1 improved high glucose-evoked insulin resistance in HepG2 cells by alleviating inflammation and lipotoxicity through. AHR activation. Appl. Biochem. Biotechnol. 195, 6694–6707. doi: 10.1007/s12010-023-04399-9, PMID: 36913096

[B99] Saliba A. Debnath S. Tamayo I. Lee H. J. Ragi N. Das F. . (2025). Quinolinic acid potentially links kidney injury to brain toxicity. JCI Insight 10, e180229. doi: 10.1172/jci.insight.180229, PMID: 39946208 PMC11949017

[B100] Sârb O. F. Iacobescu M. Soporan A. M. Mureșan X. M. Sârb A. D. Stănciulescu R. . (2025). Brain-gut interplay: Cognitive performance and biomarker correlations in IBD patients. J. Clin. Med. 14, 2293. doi: 10.3390/jcm14072293, PMID: 40217741 PMC11989679

[B101] Savitz J. (2020). The kynurenine pathway: A finger in every pie. Mol. Psychiatry 25, 131–147. doi: 10.1038/s41380-019-0414-4, PMID: 30980044 PMC6790159

[B102] Scott S. A. Fu J. J. Chang P. V. (2020). Microbial tryptophan metabolites regulate gut barrier function via the aryl hydrocarbon receptor. Proc. Natl. Acad. Sci. U.S.A. 117, 19376–19387. doi: 10.1073/pnas.2000047117, PMID: 32719140 PMC7431026

[B103] Sinenko S. A. Kuzmin A. A. Skvortsova E. V. Ponomartsev S. V. Efimova E. V. Bader M. . (2023). Tryptophan hydroxylase-2-mediated serotonin biosynthesis suppresses cell reprogramming into pluripotent state. Int. J. Mol. Sci. 24, 4862. doi: 10.3390/ijms24054862, PMID: 36902295 PMC10003565

[B104] Sordillo P. P. Sordillo L. A. Helson L. (2017). The kynurenine pathway: A primary resistance mechanism in patients with glioblastoma. Anticancer Res. 37, 4699–4705. doi: 10.21873/anticanres.11551, PMID: 28476779

[B105] Staats Pires A. Tan V. X. Heng B. Guillemin G. J. Latini A. (2020). Kynurenine and tetrahydrobiopterin pathways crosstalk in pain hypersensitivity. Front. Neurosci. 14, 620. doi: 10.3389/fnins.2020.00620, PMID: 32694973 PMC7338796

[B106] Stockwin L. H. Han B. Yu S. X. Hollingshead M. G. ElSohly M. A. Gul W. . (2009). Artemisinin dimer anticancer activity correlates with heme-catalyzed reactive oxygen species generation and endoplasmic reticulum stress induction. Int. J. Cancer 125, 1266–1275. doi: 10.1002/ijc.24496, PMID: 19533749 PMC2752979

[B107] Stone T. W. Williams R. O. (2023). Modulation of T cells by tryptophan metabolites in the kynurenine pathway. Trends Pharmacol. Sci. 44, 442–456. doi: 10.1016/j.tips.2023.04.006, PMID: 37248103

[B108] Szelest M. Walczak K. Plech T. (2021). A new insight into the potential role of tryptophan-derived. AHR ligands in skin physiological and pathological processes. Int. J. Mol. Sci. 22, 1104. doi: 10.3390/ijms22031104, PMID: 33499346 PMC7865493

[B109] Taleb S. (2019). Tryptophan dietary impacts gut barrier and metabolic diseases. Front. Immunol. 10, 2113. doi: 10.3389/fimmu.2019.02113, PMID: 31552046 PMC6746884

[B110] Tanaka M. Szabó Á Spekker E. Polyák H. Tóth F. Vécsei L. (2022). Mitochondrial impairment: A common motif in neuropsychiatric presentation? The link to the tryptophan-kynurenine metabolic system. Cells 11, 2607. doi: 10.3390/cells11162607, PMID: 36010683 PMC9406499

[B111] Tanaka M. Tóth F. Polyák H. Szabó Á Mándi Y. Vécsei L. (2021). Immune influencers in action: Metabolites and enzymes of the tryptophan-kynurenine metabolic pathway. Biomedicines 9, 734. doi: 10.3390/biomedicines9070734, PMID: 34202246 PMC8301407

[B112] Thakur A. Qiu G. Xu C. Han X. Yang T. Ng S. P. . (2020). Label-free sensing of exosomal MCT1 and CD147 for tracking metabolic reprogramming and Malignant progression in glioma. Sci. Adv. 6, eaaz6119. doi: 10.1126/sciadv.aaz6119, PMID: 32637597 PMC7319757

[B113] Tufail M. Jiang C. H. Li N. (2024). Altered metabolism in cancer: insights into energy pathways and therapeutic targets. Mol. Cancer 23, 203. doi: 10.1186/s12943-024-02119-3, PMID: 39294640 PMC11409553

[B114] Untereiner A. A. Wang R. Ju Y. Wu L. (2016). Decreased gluconeogenesis in the absence of cystathionine gamma-lyase and the underlying mechanisms. Antioxid Redox Signal 24, 129–140. doi: 10.1089/ars.2015.6369, PMID: 26401978 PMC4742978

[B115] Vaidyanathan B. Chaudhry A. Yewdell W. T. Angeletti D. Yen W. F. Wheatley A. K. . (2017). The aryl hydrocarbon receptor controls cell-fate decisions in B cells. J. Exp. Med. 214, 197–208. doi: 10.1084/jem.20160789, PMID: 28011866 PMC5206498

[B116] Venkatesan D. Arul Narayanasamy M. I. Siva K. Vellingiri B. (2020). Kynurenine pathway in Parkinson’s disease—an update. ENeurologicalsci 21, 100270. doi: 10.1016/j.ensci.2020.100270, PMID: 33134567 PMC7585940

[B117] Vogel C. F. Goth S. R. Dong B. Pessah I. N. Matsumura F. (2008). Aryl hydrocarbon receptor signaling mediates expression of indoleamine 2,3-dioxygenase. Biochem. Biophys. Res. Commun. 375, 331–335. doi: 10.1016/j.bbrc.2008.07.156, PMID: 18694728 PMC2583959

[B118] Wadan A. H. S. Shaaban A. H. El-Sadek M. Z. Mostafa S. A. Moshref A. S. El-Hussein A. . (2025). Mitochondrial-based therapies for neurodegenerative diseases: a review of the current literature. Naunyn Schmiedebergs Arch. Pharmacol. 398, 11357–11386. doi: 10.1007/s00210-025-04014-0, PMID: 40163151 PMC12449425

[B119] Wang M. Guo J. Hart A. L. Li J. V. (2023). Indole-3-Aldehyde reduces inflammatory responses and restores intestinal epithelial barrier function partially via aryl hydrocarbon receptor(.AHR) in experimental colitis models. J. Inflammation Res. 16, 5845–5864. doi: 10.2147/JIR.S432747, PMID: 38084103 PMC10710743

[B120] Wang J. Hao Y. Yang Y. Zhang Y. Xu C. Yang R. (2025). Gut microbiota derived indole-3-acetic acid ameliorates precancerous inflammatory intestinal milieu to inhibit tumorigenesis through IL-35. J. Immunother. Cancer 13, e011155. doi: 10.1136/jitc-2024-011155, PMID: 40274281 PMC12020765

[B121] Wang Y. He F. Feng F. Liu X.-W. Dong G.-Y. Qin H. . (2010). Notch signaling determines the M1 versus M2 polarization of macrophages in antitumor immune responses. Cancer Res. 70, 4840–4849. doi: 10.1158/0008-5472.CAN-10-0269, PMID: 20501839

[B122] Wang B. Hu S. Teng Y. Chen J. Wang H. Xu Y. . (2024). Current advance of nanotechnology in diagnosis and treatment for Malignant tumors. Signal Transduct Tgt Ther. 9, 200. doi: 10.1038/s41392-024-01889-y, PMID: 39128942 PMC11323968

[B123] Wang Y. Leak R. K. Cao G. (2022). Microglia-mediated neuroinflammation and neuroplasticity after stroke. Front. Cell Neurosci. 16, 980722. doi: 10.3389/fncel.2022.980722, PMID: 36052339 PMC9426757

[B124] Wang Z. Xie X. Xue Y. Chen Y. (2025). Tryptophan-2,3-dioxygenase as a therapeutic target in digestive system diseases. Biology 14, 295. doi: 10.3390/biology14030295, PMID: 40136551 PMC11939885

[B125] Wang K. Zhang X. Li A. Qiao X. Xu Y. (2025). The mechanism of action and therapeutic potential of tumor-associated macrophages in tumor immune evasion. Front. Immunol. 16, 1545928. doi: 10.3389/fimmu.2025.1545928, PMID: 40330472 PMC12052954

[B126] Wu R. Li B. Su R. Liu X. Gao A. Luo J. . (2025). Serum tryptophan-kynurenine metabolites served as biomarkers of disease activity in rheumatoid arthritis and linked to immune imbalance. Arthritis Res. Ther. 27, 136. doi: 10.1186/s13075-025-03596-7, PMID: 40618074 PMC12228287

[B127] Wu H. Q. Pereira E. F. R. Bruno J. P. Pellicciari R. Albuquerque E. X. Schwarcz R. (2010). The astrocyte-derived α7 nicotinic receptor antagonist kynurenic acid controls extracellular glutamate levels in the prefrontal cortex. J. Mol. Neurosci. 40, 204–210. doi: 10.1007/s12031-009-9235-2, PMID: 19690987 PMC3929341

[B128] Wu C. Spector S. A. Theodoropoulos G. Nguyen D. J. M. Kim E. Y. Garcia A. . (2023). Dual inhibition of IDO1/TDO2 enhances anti-tumor immunity in platinum-resistant non-small cell lung cancer. Cancer Metab. 11, 7. doi: 10.1186/s40170-023-00307-1, PMID: 37226257 PMC10207715

[B129] Xia Y. Shi C. Lu J. Zhu Z. Li M. Pan Y. . (2025). Artemisinin and its derivatives from molecular mechanisms to clinical applications: new horizons beyond antimalarials. Int. J. Mol. Sci. 26, 8409. doi: 10.3390/ijms26178409, PMID: 40943352 PMC12429344

[B130] Xu B. Fu Y. Yin N. Qin W. Huang Z. Xiao W. . (2024). Bacteroides thetaiotaomicron and Faecalibacterium prausnitzii served as key components of fecal microbiota transplantation to alleviate colitis. Am. J. Physiol. Gastrointest Liver Physiol. 326, G607–G621. doi: 10.1152/ajpgi.00303.2023, PMID: 38502145 PMC11376976

[B131] Xia H. Ke Y. Liao R. Zhang H. (2025a). Fractional order dung beetle optimizer with reduction factor for global optimization and industrial engineering optimization problems. Artificial Intelligence Review 58, 308. doi: 10.1007/s10462-025-11239-1

[B132] Xia H. Xia H. Huang J. (2025b). An Improved Machine Learning Model for Screening and Activity Prediction of Receptor Tyrosine Kinase. Journal of Bionic Engineering. doi: 10.1007/s42235-025-00816-3

[B133] Xia H. Sun L. Xia H. Seyedali M. (2025c). 3D path planning in complex mountainous environments for UAVs using quaternion-based dung beetle optimizer. Cluster Computing 28, 935. doi: 10.1007/s10586-025-05316-x

[B134] Xia H. Chen L. Xu H. (2025d). Multi-strategy dung beetle optimizer for global optimization and feature selection. International Journal of Machine Learning and Cybernetics 16, 189–231. doi: 10.1007/s13042-024-02197-1

[B135] Yi C. Huang S. Zhang W. Guo L. Xia T. Huang F. . (2025). Synergistic interactions between gut microbiota and short chain fatty acids: Pioneering therapeutic frontiers in chronic disease management. Microb. Pathog. 199, 107231. doi: 10.1016/j.micpath.2024.107231, PMID: 39681288

[B136] Zelante T. Iannitti R. G. Cunha C. De Luca A. Giovannini G. Pieraccini G. . (2013). Tryptophan catabolites from microbiota engage aryl hydrocarbon receptor and balance mucosal reactivity via interleukin-22. Immunity 39, 372–385. doi: 10.1016/j.immuni.2013.08.003, PMID: 23973224

[B137] Zelic M. Pontarelli F. Woodworth L. Zhu C. Mahan A. Ren Y. . (2021). RIPK1 activation mediates neuroinflammation and disease progression in multiple sclerosis. Cell Rep. 35, 109112. doi: 10.1016/j.celrep.2021.109112, PMID: 33979622 PMC8917516

[B138] Zhao R. Z. Jiang S. Zhang L. Yu Z. B. (2019). Mitochondrial electron transport chain, ROS generation and uncoupling. Int. J. Mol. Med. 44, 3–15. doi: 10.3892/ijmm.2019.4188, PMID: 31115493 PMC6559295

[B139] Zhao L. Suolang Y. Zhou D. Tang Y. Zhang Y. (2018). Bifidobacteria alleviate experimentally induced colitis by upregulating indoleamine 2,3-dioxygenase expression. Microbiol. Immunol. 62, 71–79. doi: 10.1111/1348-0421.12562, PMID: 29226383

[B140] Zheng W. Liu M. Lv X. He C. Yin J. Ma J. (2025). AhR governs lipid metabolism: the role of gut microbiota. Front. Microbiol. 16, 1442282. doi: 10.3389/fmicb.2025.1442282, PMID: 39944639 PMC11817270

[B141] Zheng Y. Wei K. Jiang P. Zhao J. Shan Y. Shi Y. . (2024). Macrophage polarization in rheumatoid arthritis: signaling pathways, metabolic reprogramming, and crosstalk with synovial fibroblasts. Front. Immunol. 15, 1394108. doi: 10.3389/fimmu.2024.1394108, PMID: 38799455 PMC11116671

[B142] Zhu Y. Jiang C. Liu Y. Li Y. Wu H. Feng J. . (2020). Association between IDO activity and prognosis in patients with non-small cell lung cancer after radiotherapy. Ann. Transl. Med. 8, 1169. doi: 10.21037/atm-20-5634, PMID: 33241018 PMC7576049

[B143] Zmarowski A. Wu H. Q. Brooks J. M. Potter M. C. Pellicciari R. Schwarcz R. . (2009). Astrocyte-derived kynurenic acid modulates basal and evoked cortical acetylcholine release. Eur. J. Neurosci. 29, 529–538. doi: 10.1111/j.1460-9568.2008.06594.x, PMID: 19187269

